# Phenotypical Characterization of the Nuclear Egress of Recombinant Cytomegaloviruses Reveals Defective Replication upon ORF-UL50 Deletion but Not pUL50 Phosphosite Mutation

**DOI:** 10.3390/v13020165

**Published:** 2021-01-22

**Authors:** Sigrun Häge, Eric Sonntag, Adriana Svrlanska, Eva Maria Borst, Anne-Charlotte Stilp, Deborah Horsch, Regina Müller, Barbara Kropff, Jens Milbradt, Thomas Stamminger, Ursula Schlötzer-Schrehardt, Manfred Marschall

**Affiliations:** 1 Institute for Clinical and Molecular Virology, Friedrich-Alexander University of Erlangen-Nürnberg (FAU), 91054 Erlangen, Germany; sigrun.haege@fau.de (S.H.); ericsonntag@web.de (E.S.); adriana.svrlanska@gmx.de (A.S.); de.horsch@gmx.de (D.H.); mueller.regina@uk-erlangen.de (R.M.); barbara.kropff@uk-erlangen.de (B.K.); jens.milbradt@lgl.bayern.de (J.M.); 2 Institute of Virology, Hannover Medical School (MHH), 30625 Hannover, Germany; borst.eva@mh-hannover.de; 3 Institute for Virology, Ulm University Medical Center, 89081 Ulm, Germany; anne-charlotte.stilp@uniklinik-ulm.de (A.-C.S.); thomas.stamminger@uniklinik-ulm.de (T.S.); 4 Department of Ophthalmology, University Medical Center Erlangen (FAU), 91054 Erlangen, Germany; Ursula.Schloetzer-Schrehardt@uk-erlangen.de

**Keywords:** human cytomegalovirus, core nuclear egress complex, ORF-UL50 deletion, pUL50 phosphosite mutants, phenotypical changes, differential functional relevance

## Abstract

Nuclear egress is a common herpesviral process regulating nucleocytoplasmic capsid release. For human cytomegalovirus (HCMV), the nuclear egress complex (NEC) is determined by the pUL50-pUL53 core that regulates multicomponent assembly with NEC-associated proteins and capsids. Recently, NEC crystal structures were resolved for α-, β- and γ-herpesviruses, revealing profound structural conservation, which was not mirrored, however, by primary sequence and binding properties. The NEC binding principle is based on hook-into-groove interaction through an N-terminal hook-like pUL53 protrusion that embraces an α-helical pUL50 binding groove. So far, pUL50 has been considered as the major kinase-interacting determinant and massive phosphorylation of pUL50-pUL53 was assigned to NEC formation and functionality. Here, we addressed the question of phenotypical changes of ORF-UL50-mutated HCMVs. Surprisingly, our analyses did not detect a predominant replication defect for most of these viral mutants, concerning parameters of replication kinetics (qPCR), viral protein production (Western blot/CoIP) and capsid egress (confocal imaging/EM). Specifically, only the ORF-UL50 deletion rescue virus showed a block of genome synthesis during late stages of infection, whereas all phosphosite mutants exhibited marginal differences compared to wild-type or revertants. These results (i) emphasize a rate-limiting function of pUL50 for nuclear egress, and (ii) demonstrate that mutations in all mapped pUL50 phosphosites may be largely compensated. A refined mechanistic concept points to a multifaceted nuclear egress regulation, for which the dependence on the expression and phosphorylation of pUL50 is discussed.

## 1. Introduction

Human cytomegalovirus (HCMV) represents a ubiquitous pathogen that causes severe sequelae in immunocompromised patients and newborns. During lytic replication, HCMV hijacks cellular processes to allow the efficient progression of its lytic replication cycle. A crucial step is the well-orchestrated transition process of viral capsids through the nuclear envelope. Hereby, viral capsids have to overcome the nuclear lamina, a proteinaceous meshwork that limits viral budding through the inner and outer nuclear membranes. HCMV-induced disassembly of the nuclear lamina is mediated by a defined nuclear egress complex (NEC) that associates both viral and host proteins [1]. Two virus-encoded NEC proteins have homologs in all known herpesviruses and have been considered as functionally essential for a balanced regulation of herpesviral nuclear capsid egress [1,2,3,4,5]. This core NEC, i.e., HCMV pUL50-pUL53, provides a scaffold that is crucial for NEC multicomponent protein assembly, capsid binding and a multifaceted functionality. On this basis, at least three main functions of the multicomponent NEC have been analyzed on a molecular basis so far: (i) to mediate the multimeric recruitment of NEC-associated effectors, (ii) to promote the reorganization of the nuclear lamina as well as membranes, and (iii) to provide a docking platform for nuclear capsids. As far as the latter aspect is concerned, we identified by immuno-gold electron microscopy (EM) a high portion of nuclear capsids being associated with pUL53 [6]. It appears generally accepted that both viral and cellular protein kinases are responsible for site-specific phosphorylation of the core NEC proteins themselves as well as of nuclear lamins and lamina-associated proteins. Recent studies demonstrated that HCMV pUL50-pUL53 core NEC formation at the nuclear rim depends on the activity of the viral kinase pUL97 [7] and additionally on cellular protein kinases such as CDK1 [8]. Of note, phosphorylation-specific mass spectrometry analysis revealed five major and nine minor phosphosites in pUL50 [8], but their functional importance remained elusive so far.

The property of being phosphorylated by viral and cellular protein kinases is shared by all analyzed members of the core NEC gene family [1,3,9]. Moreover, several studies revealed strongly conserved structural features of core NECs, particularly comparing the 3D crystal structures of herpes simplex virus type 1 (HSV-1), pseudorabies virus (PrV), HCMV, murine cytomegalovirus (MCMV) and Epstein-Barr virus [10,11,12,13,14,15,16]. Although core NEC homologs only share low to moderate degrees of sequence identity, their globular structures are remarkably conserved [3], thus comprising (i) a hook-like N-terminal heterodimerization domain in pUL53 homologs, (ii) a C-terminal α-helical binding groove in pUL50 homologs and (iii) a pUL53-specific zinc finger. Most strikingly, a common hook-into-groove principle of core NEC heterodimerization has been identified for α-, β- and γ-herpesviruses [1,3,5,9,10,14]. However, limited data are available addressing the question whether core NEC homologs are strictly functionally conserved [1,17] or whether they may display virus-specific differences. As far as the latter point is concerned, recent investigations revealed that binding properties of core NEC proteins in terms of nonautologous heterodimeric interactions are limited within herpesviral subfamilies [2,16,17], whereas those in terms of multimeric interaction with additional NEC-associated proteins can differ substantially [1]. Using bacterial artificial chromosome-derived (BAC) virus recombinants carrying NEC gene exchanges, HCMV pUL50 and pUL53 were able to rescue viral replication of MCMV mutants defective in the expression of NEC proteins in vitro [17] or in vivo [2].

Our recent analyses of NEC protein-protein interaction and phosphorylation-specific mass spectrometry pointed to pUL50 representing the multi-interacting, phospho-regulated NEC determinant [8,18]. Since the question about functional relevance of core NEC protein phosphorylation has not been fully answered, the impact of alterations in the phosphosite patterns on viral nuclear egress should be investigated. In this study, we addressed these central questions by the use of an ORF-UL50 deletion virus, using pUL50-complementing cells for virus reconstitution, as well as by introducing pUL50 phosphosite replacement mutations. The experimental design covered an analysis of viral protein expression patterns, investigation of alternative phosphosites, coimmuno-precipitation (CoIP) analysis of altered protein-protein interactions, confocal imaging of NEC protein localization and EM as well as qPCR methods to characterize parameters of viral replication. Profound functional differences were seen between the ORF-UL50 deletion virus and the recombinants harboring phosphosite mutations and their relevance for the mechanistic understanding of herpesviral NECs is discussed.

## 2. Materials and Methods

### 2.1. Cell Culture, Virus Infection and Transient Transfection

Primary human foreskin fibroblasts (HFFs, own repository of primary cell cultures) and HFF-UL50 [19] were propagated as described previously [8,19]. HeLa and 293T cells were cultivated at 37 °C, 5% CO_2_ and 80% humidity using Dulbecco’s modified Eagle’s medium (DMEM, 11960044, Thermo Fisher Scientific, Waltham, MA, USA) supplemented with 1× GlutaMAX™ (35050038, Thermo Fisher Scientific), 10 μg/mL gentamicin (22185.03, SERVA, Heidelberg, Germany) and 10% fetal bovine serum (FBS, F7524, Sigma Aldrich, St. Louis, MO, USA). HCMV strain AD169 variant UK, HCMV ΔUL50 (described in [19]) and phosphosite mutants generated in this study were propagated as stocks and titrated in the respective cell lines as described previously [20,21]. Infection experiments were performed at indicated multiplicity of infection (MOI) using parental or recombinant HCMVs. After incubation for 90 min at 37 °C, virus inoculi were removed and replaced by fresh growth medium. Transient transfection of expression plasmids was performed as described before [2].

### 2.2. Plasmids

For the generation of recombinant HCMVs, universal transfer constructs (UTCs) carrying phosphosite mutations on the template UL50-HA were generated in vector pcDNA3.1. In addition to the wild-type-like revertant pcDNA3.1-UL50(Rev1)-HA, major phosphosites (S188, T214, S216, S300 and S324) were substituted with alanine resulting in pcDNA3.1-UL50(P×5)-HA or pcDNA3.1-UL50(S216A)-HA or with glutamic acid resulting in pcDNA3.1-UL50(M×5)-HA by site-directed mutagenesis (GeneArt™ Site-Directed Mutagenesis System, Thermo Fisher Scientific). An I-*SceI-aphAI* cassette (kanamycin cassette) from plasmid pEP-*S/aphAI* (kindly provided by B. K. Tischer; [22]) was inserted into wild-type or mutated UL50-HA UTCs via the *AflII* restriction site (Figure S1). UTCs harboring mutations at 14 phosphorylation sites (S95, T174, T183, S188, T214, S216, S225, S227, T229, S251, S268, S300, S324 and S330) were constructed by ShineGene Molecular Biotech Inc., Shanghai, China. Expression plasmids coding for pUL50-HA, pUL53-Flag, PKCα-Flag and p32(50-282)-Flag were described previously [23,24,25].

### 2.3. Generation of Recombinant HCMV

For the generation of HCMV UL50 phosphosite mutations, the BAC HB15/AD169ΔUL50 (described in [19]) (Figure S1A) was used for the insertion of UL50 phosphosite UTCs by a two-step markerless Red recombination system as described previously (Figure S1C; [19,26]). In a first step the pUL50 phosphosite UTCs were inserted into HB15/AD169ΔUL50 as described before and in antisense direction (Figure S1C; [22,26,27]). During the second recombination step, the coding sequence for the resistance cassette was deleted resulting in the recombinant BACs (Figure S1B). Recombinant viruses were reconstituted by transfection of the generated BACs into HFFs. The ΔUL50 genome, which did not yield progeny virus after transfection into normal HFFs, could be successfully used for the reconstitution of rescue virus by the generation of pUL50-complementing cells as described earlier [19]. Virus stock was grown in doxycylin-induced (+Dox), pUL50-complementing cells and was then used for infection experiments in cultures of induced, uninduced HFF-UL50 or normal HFFs. The correctness of gene sequences and the inserted mutations was verified by repeated sequence analyses of PCR fragments derived from both, templates of BAC DNA as well as reconstituted virion DNA (summarized in Figure S7).

### 2.4. Antibodies

Antibodies used in this study are mAb-IE1p72, mAb-MCP, mAb-pp28 (all kindly provided by William Britt, University of Alabama, Birmingham, AL, USA), mAb-UL44 (kindly provided by Bodo Plachter, University of Mainz, Mainz, Germany), mAb-UL50.01, mAb-UL53.01, mAb-UL97.01 (all kindly provided by Stipan Jonjic and Tihana Lenac Rovis, University of Rijeka, Rijeka, Croatia), mAb-β-Actin (A5441, Sigma Aldrich, St. Louis, MO, USA), mab-lamin A/C (ab108595, Abcam, Cambridge, UK), pAb-lamin A/C pSer22 (ABIN1532183, Antibodies online, Aachen, Germany), pAb-Pin1 (10495-1-AP, Proteintech, Rosemont, IL, USA), anti-Cytomegalovirus-Alexa Fluor 488 (IE1/MAB810X, Merck, Darmstadt, Germany), mAb-HA (Clone 7, H9658, Sigma Aldrich), mAb-Flag (F1804, Sigma Aldrich), mAb-emerin (Sc-25284, Santa Cruz, Dallas, TX, USA), anti-mouse Alexa 555 (A-21422, Thermo Fisher Scientific, Waltham, MA, USA) and anti-rabbit Alexa 488 (A-11008, Thermo Fisher Scientific).

### 2.5. CoIP, Phosphate Affinity (Phos-Tag) SDS-PAGE and Western Blot (Wb) Analyses

For the investigation of expression patterns, HCMV-infected HFFs were harvested and lysed at the time points indicated. To analyze protein-protein interactions in a transient expression system, 293T cells were seeded into 10-cm dishes with a density of 5.0 × 10^6^ cells and transfected with expression plasmids. Three days post transfection (d p.t.), CoIP was performed as described previously [18]. Immunoprecipitation control samples of approximately one-tenth of total volumes were taken prior to CoIP reactions. Samples were subjected to SDS–PAGE/Wb procedure as described previously [28]. For the identification of phosphorylated protein varieties, HFFs were infected with HCMVs at an MOI of 0.3, harvested 4 days post infection (d.p.i). and lysed in CoIP buffer without EDTA for 20 min on ice. For lambda phosphatase (PP) treatment, samples were supplemented with 1 × PMP buffer, 1 mM MnCl_2_ and 0.5 μL of lambda phosphatase (P0753S, New England Biolabs, Ipswich, MA, USA), incubated at 30 °C for 45 min and subsequently denatured at 95 °C for 10 min. Samples were analyzed by standard SDS-PAGE, supplemented with Phos-tag reagent [29] (AAL-107, Wako PureChemical Industries, Osaka, Japan) according to manufacturer’s instructions, and a subsequent Wb immunostaining.

### 2.6. Indirect Immunofluorescence Assay and Confocal Laser-Scanning Microscopy

Transiently transfected HeLa cells or HCMV-infected HFFs were grown on coverslips, fixed at 2 d p.t. or 6 d p.i. with 10% formalin solution (10 min, room temperature) and permeabilized by incubation with 0.2% Triton X-100 solution (15 min, 4 °C). Indirect immunofluorescence staining was performed by incubation with primary antibodies as indicated for 60 min at 37 °C, followed by incubation with dye-conjugated secondary antibodies for 30 min at 37 °C. Cells were mounted with Vectashield Mounting Medium containing DAPI (H-1700, Vector Laboratories, Burlingame, CA, USA) and analyzed using a TCS SP5 confocal laser-scanning microscope (Leica Microsystems, Wetzlar, Germany). Images were processed using the LAS AF software (Leica Microsystems) and Photoshop CS5 (Adobe Inc., San José, CA, USA).

### 2.7. Cytomegalovirus Multistep Replication Curve Analysis

Infection experiments were performed at indicated MOIs using parental or recombinant HCMVs. Viral replication kinetics were analyzed by quantitative real-time PCR (qPCR) or digital droplet PCR (ddPCR) as described previously [21,30]. In the ddPCR procedure, DNA samples are individually fractionated in 20,000 droplets, each droplet containing either no template, one template or more templates. Thus, ddPCR is a binary end-point measurement resulting in either a positive or a negative signal [31,32]. On this basis, one single copy can be detected, so that per definition the ddPCR limit of detection was set as 10^0^ copies. The standard containing 10 HCMV DNA copies reached the cycle threshold at cycle 38 and was therefore defined as the limit of detection for qPCR.

### 2.8. Transmission Electron Microscopy (TEM)

HFFs were plated on Nunc™ Thermanox™ coverslips (174977, Thermo Fisher Scientific) with a density of 3.0–4.0 × 10^5^ and infected with parental or recombinant HCMVs at indicated MOI. At 4 (phosphosite mutants) or 7 d p.i. (ΔUL50), cells were fixed with 2.5% glutaraldehyde in 0.1M phosphate buffer, postfixed in 2% buffered osmium tetroxide, dehydrated in graded alcohol concentrations, and embedded in epoxy resin according to standard protocols. Ultrathin sections were stained with uranyl acetate and lead citrate and examined with a transmission electron microscope (LEO 906E, Carl Zeiss Microscopy GmbH, Oberkochen, Germany).

## 3. Results

### 3.1. Construction of Expression Plasmids and Recombinant Viruses Encoding Mutant Versions of ORF-UL50

**Figure 1:**
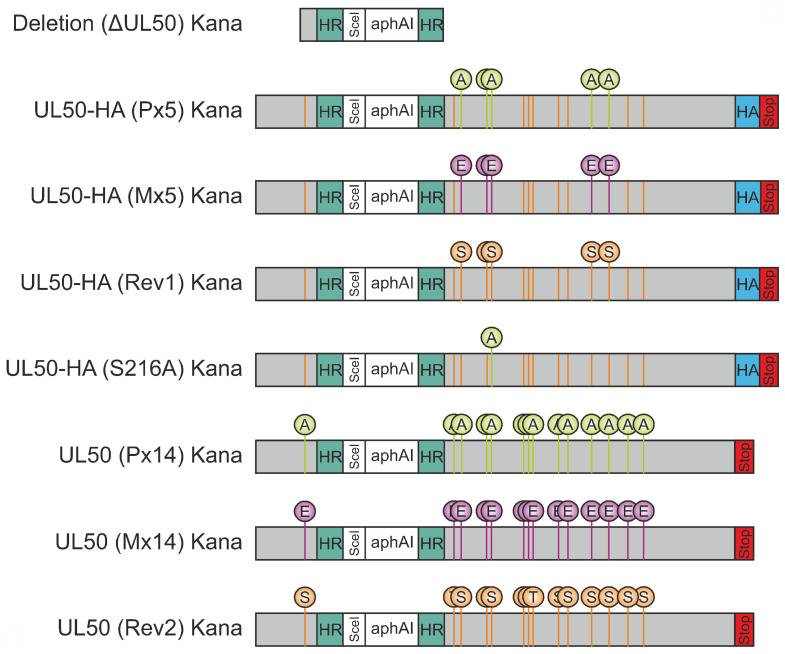
Schematic view of ORF-UL50 constructs used in this study. For the construct termed ‘Deletion (ΔUL50) Kana’, ORF-UL50 was deleted from the HB15 BACmid using Red recombination without affecting the pUL49/pUL50 overlapping region (nt 1151–1194 highlighted in light grey; [19]). The construction of universal transfer constructs (UTCs, inserted into pcDNA3.1 plasmid vector) was based on wild-type UL50-HA, containing the homologous regions (HR) required for Red recombination, an I-SceI restriction site and a kanamycin cassette (aphAI). The serine/threonine replacement mutants (five major phosphosites S188, T214, S216, S300, S324 and nine minor phosphosites S95, T174, T183, S225, S227, S229, S251, S268, S330), as generated by site-directed mutagenesis, were termed as P×5 and P×14 (5 or 14 alanine replacement mutations) or M×5 and M×14 (5 or 14 phosphorylation mimetic mutations, carrying glutamic acid replacements at identical positions). Two corresponding revertants, termed Rev1 and Rev2, were used as control constructs for the P×5/M×5 and P×14/M×14 mutants, respectively, and a single-site mutant, S216A, was additionally produced for comparison. HA-tag is highlighted in blue and stop codons in red. Details for BAC recombination using these UTCs is described by Figure S1.

For the analysis in various expression and infection systems, a series of constructs was generated. Transient expression was used as a basis for Wb detection of mutant pUL50, CoIP experiments and confocal immunofluorescence imaging. To this end, plasmid constructs were generated by cloning WT ORF-UL50-HA into vector pcDNA3.1 and subsequent site-directed mutagenesis to produce replacement mutations in all 14 phosphosites reported in our earlier study [8]. These phosphosites, i.e., serine or threonine residues, were categorized in five major (S188, T214, S216, S300 and S324) and nine minor sites (S95, T174, T183, S225, S227, S229, S251, S268 and S330) on the basis of their abundance of detectability by phosphorylation-specific mass spectrometry analyses. Replacement mutations were either performed through alanine (loss-of function, P×5 and P×14) or glutamic acid exchanges (gain-of-function, M×5 and M×14). The latter mutants were intended to mimic the phosphorylated state on the basis of the amino acids negative charge. Revertants to the original phosphosite pattern were also produced in the five major positions (Rev1) or all 14 positions (Rev2). Recombinant viruses were generated using two-step traceless BAC mutagenesis of the HB15 genome referring to the HCMV laboratory strain AD169 [22]. The constructs and procedures are described in Figure 1 and Figure S1. Sequencing of relevant regions was performed, so that the correctness of ORF-UL50 deletion and phosphosite mutations in the respective BACs as well as the genomes of reconstituted viruses could be verified. In order to address the question of putative compensatory mutations in other, functionally relevant viral coding sequences, also ORFs UL53 and UL97 were included in this analysis. With the exception of one identical amino acid replacement in P×5, M×5 and Rev1, no additional mutations were detected (Figure S7). Thus, we did not find an indication of a selective pressure towards compensatory mutations induced by ORF-UL50 mutagenesis in these virus recombinants.

### 3.2. Patterns of Expression and Protein-Protein Interaction of ORF-UL50 Deletion and Phosphosite Mutants

**Figure 2:**
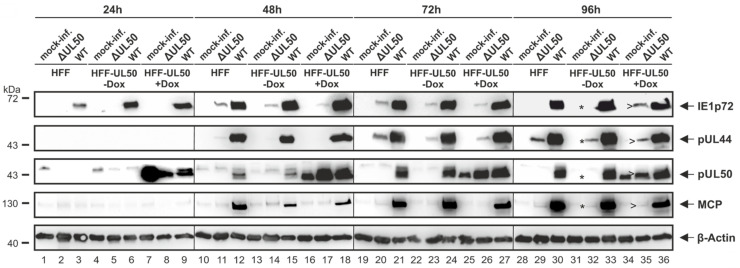
Analysis of viral protein expression of recombinant HCMV ΔUL50. Normal HFFs were grown in 6-well plates (4 × 10^5^ cells/well) and used for the production of primary infectious supernatants of parental HCMV AD169 (WT) and recombinant HCMV ΔUL50. These supernatants were used as inocula (identical volumes of 1 mL/well) for the infection of normal HFF or HFF-UL50 −Dox/+Dox cells as indicated, to determine the expression patterns of HCMV WT in comparison to ΔUL50. Protein expression was analyzed at the time points indicated (h p.i.) and Western blotting (Wb) was performed using the respective antibodies. Note the marked quantitative differences, i.e., the low level of infectivity and viral protein production, even after 96 h p.i., obtained with the ΔUL50 inoculum, particularly in uninduced (*, −Dox) compared to pUL50-induced cells (>, +Dox).

In order to analyze the regulatory relevance of deletion and phosphosite mutations, patterns of viral protein expression were determined under various conditions. The rescue virus of HCMV ΔUL50 was used for infection experiments performed in pUL50-complementing cells, i.e., the HFF-UL50 cells induced with doxycyclin (+Dox; [19]), in parallel to pUL50-negative HFF-UL50 (−Dox) or normal HFFs (Figure 2). The impact of +Dox induction on HFF-UL50 cells for successful pUL50 complementation and rescue of the defective ΔUL50 phenotype had been described before [19]. In the present experiment, infection of the different cells (HFF-UL50+Dox, −Dox and normal HFFs) was performed with infectious supernatant samples freshly produced under the following conditions: normal HFFs were used for the production of primary infectious supernatants of parental HCMV AD169 (WT) and recombinant HCMV ΔUL50. The supernatants were harvested at the time point when virus-induced cytopathic effect had occurred in approximately 50% of the monolayers. These supernatants were then used as inocula for the infection of normal HFF or HFF-UL50−Dox/+Dox cells to determine the differential viral expression patterns. Note the marked quantitative differences of infectivity of ΔUL50 virus compared to WT (Figure 2). Cells were harvested at consecutive time points between 24 and 96 h p.i. (h p.i.) and viral proteins of all replicative stages, i.e., immediate early (IE1p72), early (pUL44 and pUL50) and late (major capsid protein, MCP), were detected by Wb analysis. Notably, HCMV ΔUL50 showed a delay and massive quantitative reduction in the production of viral proteins, but surprisingly late-period expression was not completely abolished. Note the detectable signals of IE1p72 and pUL44 expression at time points of 48–72 h p.i. or 72–96 h p.i., respectively (Figure 2, lanes 11, 14, 20, 23, 29 and 32). It was also striking to find a very low level or even the absence of expression of the true-late protein MCP for HCMV ΔUL50. In addition, a lack of continuous presence of IE1p72 was noted at late time points of infection in the absence of Dox (96 h p.i., upper panel). Thus for the ΔUL50 virus, a very low level of infectivity and viral protein production was observed compared to WT, particularly close to the detection limit in uninduced cells (uninduced *, −Dox; induced >, +Dox). This difference was detectable for immediate early, early as well as late proteins. The finding strongly suggested that the ΔUL50 mutant, although not being entirely defective in the production of viral proteins, showed a limited and transient mode of immediate early/early protein expression. Infection periods later than 96 h were analyzed by PCR-based measurement of viral replication kinetics as described below (up to 14 d p.i.).

**Figure 3:**
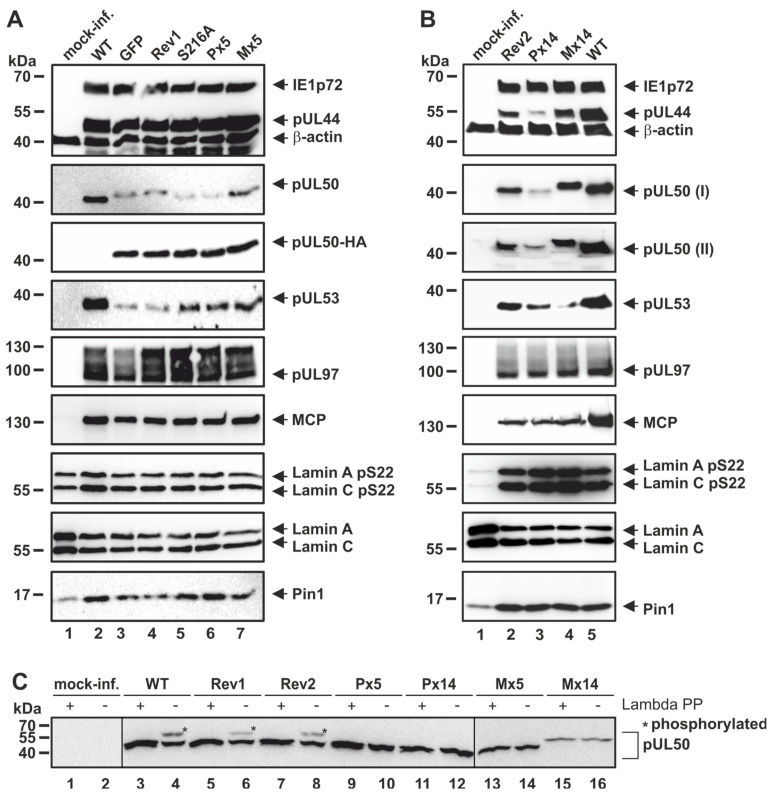
Analysis of viral protein expression of HCMV pUL50 phosphosite mutants. HFFs were infected with parental or recombinant HCMVs at a MOI of 0.3. Protein expression was analyzed after 4 d p.i. and Wb was performed using the indicated antibodies. (**A**) Focus was given either to major phosphosite mutants P×5 and M×5, or (**B**) the mutants including major plus minor phosphosite replacements in P×14 and M×14. Note the reduced pUL53 and pUL50 levels, as explained in the text, the latter depicted by two independent experimental replicates, labeled pUL50 (I) and (II). (**C**) Total lysates of HCMV-infected HFFs were optionally treated with lambda phosphatase (PP) for 45 min at 30 °C and subjected to Phos-tag SDS-PAGE and Wb; *, phosphorylated form of pUL50.

In contrast to the ΔUL50 virus, HCMV pUL50 phosphosite mutants (MOI of 0.3, Figure 3), showed mostly unaltered patterns of expression of viral proteins, which was basically indistinguishable from the parental WT virus (Figure 3). The expression patterns of IE1p72, pUL44, pUL50, pUL53, pUL97 and MCP, although showing some minor variations, did not reveal a drastic change for the virus recombinants P×5, M×5, S216A at 4 d p.i. (compared to WT, the GFP-expressing reporter version AD169-GFP or the ORF-UL50 revertants Rev1 or Rev2). This finding of poorly altered expression patterns was mostly consistent for all mutants, i.e., including the major phosphosite mutants (P×5, M×5; Figure 3A), the single-site mutant S216A (Figure 3A), and the minor phosphosite mutants (P×14, M×14; Figure 3B). The finding for S216A was surprising, since a previous report demonstrated a negative effect of pUL50 mutation S216A on viral nuclear egress. In the reported case, however, the mutant virus carried a second mutation, namely pUL53 S19A [7]. This suggests that S216A alone may be compensated while double-mutations in pUL50/pUL53 may produce such phenotypical effect. Interestingly in the present experiments, the phosphosite mutants showed some variation in the quantities and kinetics of individual viral proteins, including pUL53 and/or pUL50 themselves, but this could not be clearly correlated with genotypic differences. Such altered expression levels appeared to be compensated, since the overall viral replication kinetics (see below) did not indicate detectable replicative defects. In this context, it should also be mentioned that all HCMVs expressing HA-tagged pUL50 (Figure 3A, lanes 4–7) showed somewhat reduced levels of pUL50 and pUL53 compared to WT, an observation already described before [27]. Moreover, also phosphorylated (pS22) lamin A/C and Pin1, did not show a clear-cut virus-specific alteration in most cases analyzed here, and did not markedly differ from the house-keeping protein β-actin. A general phenomenon of a virus-induced effect on the cellular level, observed already earlier [33,34,35], was confirmed here, namely a slight upregulation of lamin A/C pS22 and Pin1 as well as downregulation of lamin A/C by HCMV infection. Concerning the mutants’ protein characteristics in SDS-PAGE separation, P×14 and M×14 showed pronounced faster or slower migration, respectively, when compared to WT. This was found in the settings of both recombinant viruses and transient expression (Figure 3B and Figure S2), and such altered migration behavior was in part also observed for the other mutants (Figure 3A). This may be explained by the massive loss of overall charge and the change in molecular masses upon replacement of serine/threonine residues to alanine or glutamic acid.

Given the fact that a previous publication demonstrated the functional importance of HCMV pUL50/pUL53 phosphorylation sites [7], it appeared plausible that alternative patterns of phosphorylation may replace the originally identified patterns [18]. Possibly, the phosphorylation of pUL50 may carry some intrinsic flexibility, so that the preferred positions of phosphorylation should not be as decisive as the overall negative charge of subdomains and the mimic options of alternative amino acids. Of note, HCMV pUL50 and pUL53 in total contain 59 and 42 serine/threonine residues, respectively, within their entire amino acid sequences. In order to test the possibility of alternative phosphorylation, a Phos-tag-specific Wb analysis was performed (Figure 3C). A comparison between samples of total cellular protein lysates derived from the individual virus mutants showed that the mutants P×5, M×5, P×14 and M×14 lack the phosphorylation signals (asterisks) detected for WT and Rev viruses. This may indicate that alternative pUL50 phosphorylation of the mutants was either not detectable or pUL50 phosphorylation may not be functionally important.

When analyzing the interaction capacity of the pUL50 mutants P×14 and M×14 upon transient expression, all interactions with the known interaction partners, i.e., pUL53, PKCα, p32/gC1qR and emerin, were detectable by CoIP analysis (Figure S2). Considering the quantitative levels of interaction, referring to 100% defined for pUL50-HA as the non-mutated parental protein, reduced CoIP signals were found in most cases for both of the two types of pUL50 mutants, P×14 and M×14 (Figure S2B–D; note the exception of pUL53 interaction which was found to be increased, Figure S2A).

### 3.3. Confocal Imaging of Mutant pUL50 Expressed by Plasmids or Recombinant HCMVs: Localization of Viral Proteins and Nuclear Lamina Components

**Figure 4:**
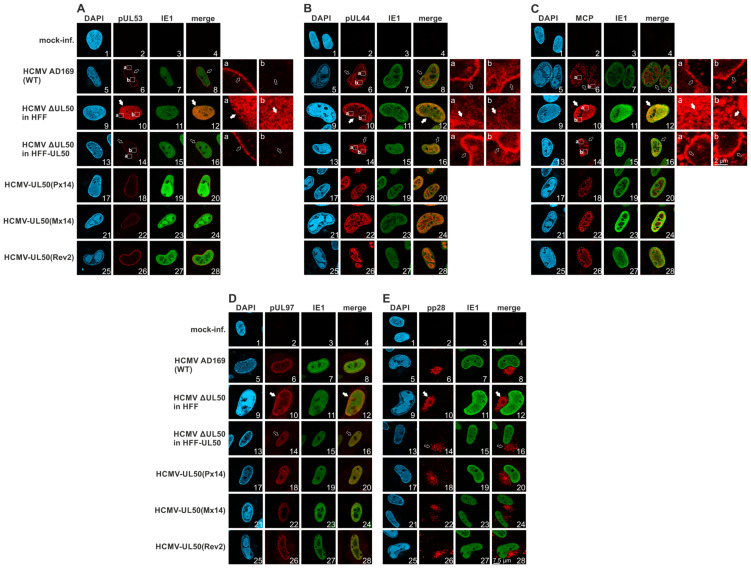
Confocal imaging analysis of the localization of viral proteins produced by HCMV ΔUL50 or pUL50 phosphosite mutations. Normal HFFs or pUL50-complementing cells (HFF-UL50 +Dox) were used for infection with the recombinant HCMVs at a MOI of 0.05 and harvested at 7 d p.i. Immunofluorescence staining was performed with antibodies against the indicated proteins and representative panels of confocal imaging are given (see scale bar in panel E, picture 28). (**A**–**C**) Viral proteins pUL53, pUL44 and MCP, showing altered localization patterns for HCMV ΔUL50 in HFF (see insets a and b and the phenotype of altered localization marked by filled white arrows, compared to normal/unaltered localization marked by framed white arrows). (**D**,**E**) Viral proteins pUL97, IE1 and pp28, showing no alteration in localization patterns. Additional information is provided by Figure S4.

The intracellular localization of nuclear egress-relevant proteins was investigated by confocal imaging, using both transiently transfected HeLa cells and HCMV-infected primary fibroblasts. Upon transient plasmid transfection of mutant pUL50, the question of relocalization of coexpressed pUL53 was specifically addressed. Generally, the single expression of pUL53 shows an even distribution throughout the nucleus (Figure S3A, panel i), whereas pUL50-pUL53 coexpression is typically characterized by a prominent nuclear rim colocalization (Figure S3A, panels d–f), so that this phenotype was analyzed for all phosphosite mutants. As a clear result, none of the mutants, P×5, M×5, P×14 or M×14, showed a substantial difference compared to the parental pUL50-HA, i.e., the relocalization of pUL53 to the nuclear rim was detected in all cases (Figure S3B,C, panels g–r). Some variations in the speckled pattern of rim formation were visible, but these were not based on mutant-specific phenotypical alterations. Next, HCMV-infected HFFs (MOI of 0.05, late-stage infection at 7 d p.i.) were analyzed by confocal imaging in an identical manner. In this case, the HCMV ΔUL50 showed very pronounced differences towards the parental WT strain: (i) pUL53 completely lacked the nuclear rim colocalization, but showed an even distribution throughout the nucleus, (ii) the viral DNA polymerase processivity factor pUL44 lacked its typical accumulation in viral nuclear replication compartments, but showed a mostly diffuse staining pattern, and (iii) MCP similarly lacked a replication compartment-specific accumulation (normally enhanced at their edge areas), but was also mostly diffuse (Figure 4A–C, upper four lines of panels). Thus, these three viral proteins were drastically delocalized in cells infected with the rescue HCMV ΔUL50. In this context, it should be emphasized that there was a clear difference between infection of normal HFF and HFF-UL50 +Dox cells in the localization patterns of these three proteins (Figure 4A–C, panels 9–16 each), concluding that the pUL50-complementing cells rescued the phenotype of the ΔUL50 mutant. Of note, other viral proteins did not show similar changes, like IE1 and pUL97 (both nuclear) or pp28 (cytoplasmic cVAC structures; Figure 4D,E, upper four lines of panels). This indicates that this mutant is able to transiently produce viral proteins belonging to all replicative stages from immediate early to late, but that the regulation of intracellular protein localization and transport is massively disturbed.

As far as cellular egress-relevant proteins were concerned, we analyzed lamin A/C, Ser22-phosphorylated lamin A/C and emerin (Figure S4A–C, upper four lines of panels). Interestingly none of these proteins showed virus-specific alterations when comparing HCMV ΔUL50 and the phosphosite mutants with the WT virus. It was striking to see that infection with P×14 and M×14 resulted in protein localization patterns, for all viral and cellular proteins analyzed, that we considered uneventful and indistinguishable from WT or the respective revertant Rev2 (Figure 4A–E and Figure S4A–C, lower three lines of panels). Combined these confocal imaging data, in the case of HCMV ΔUL50 mutant revealed marked characteristics of altered protein localization patterns, specifically for three viral proteins, but surprisingly not for the mutants carrying 14 phosphosite replacements.

### 3.4. Characteristic Differences in the Replication Kinetics between Recombinant HCMVs Carrying ORF-UL50 Deletion or pUL50 Phosphosite Mutations

**Figure 5:**
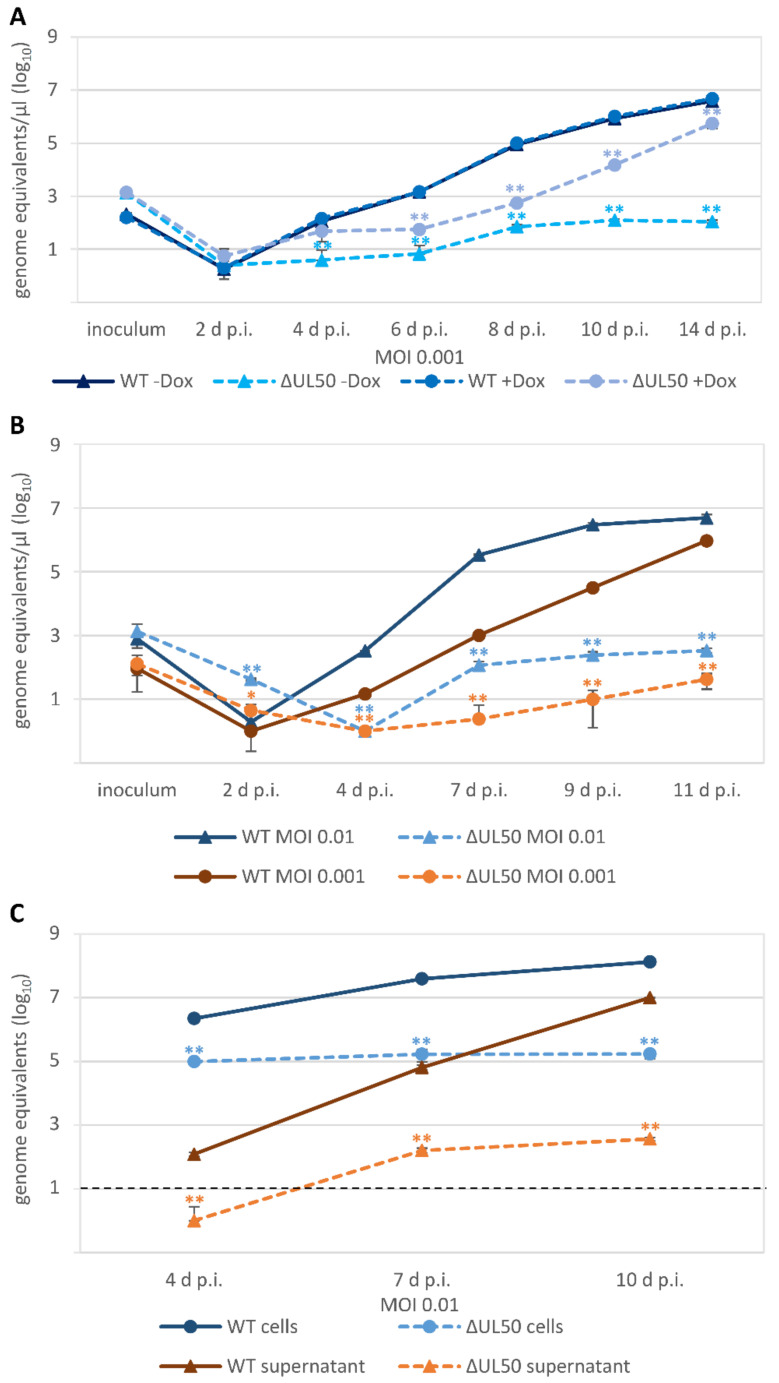
HCMV replication kinetics of the ORF-UL50 deletion mutant. HFF-UL50 cells (**A**) or HFFs (**B**,**C**) were infected with parental HCMV AD169 (WT) or recombinant HCMV ΔUL50 at a MOI of 0.01 or 0.001 as indicated. The expression of pUL50 in HFF-UL50 cells was either uninduced (−Dox) or induced (+Dox). Viral supernatants (**A**–**C**) or cells (**C**) were harvested at the indicated time points and viral genome equivalents were determined by IE1-specific ddPCR (**A**,**B**) or qPCR (**C**). Each infection was performed at least in triplicates; mean values and standard deviations are shown. The significance is calculated relating to WT (solid lines). The standard containing 10 HCMV DNA copies reached the cycle threshold at cycle 38 and was therefore defined as the limit of detection for qPCR, as shown by the black dashed line. ddPCR is a binary end-point measurement and can detect one single copy; *, *p* ≤ 0.05; **, *p* ≤ 0.01.

The investigation of viral replication kinetics revealed some unexpected findings. Firstly, we used the ΔUL50 virus and performed a multistep replication curve analysis, using both qPCR and ddPCR measurements of the same samples, derived either from infectious media supernatants or cellular lysates after HCMV infection at different MOIs. When using HFF-UL50 complementing cells, comparing the conditions of induced (+Dox, pUL50-positive) and uninduced (−Dox, pUL50-negative), no difference was observed for the parental WT by measuring supernatant samples with ddPCR. However, a clear difference between the two conditions was seen for the ΔUL50 virus (Figure 5A). The induced sample showed a strong increase in signals compared to the uninduced setting, thus indicating a rise of viral genomic load and viral replication over time. Both ddPCR curves were significantly lower than the WT reference curve. Interestingly, however, the curve of the ΔUL50 virus did not indicate a complete lack of replication, but instead showed a slight and transient increase of viral load between 2 and 10 d p.i., which came only to a plateau at later time points (14 d p.i.; Figure 5A). Data could be confirmed for these samples by using qPCR (Figure S5A,B). A very similar finding was obtained by using normal HFFs for infection, either at MOI 0.01 or 0.001 (Figure 5B, ddPCR; see reproduction of data by qPCR in Figure S5C,D), and likewise supernatants and cellular lysates from MOI 0.01 in parallel (Figure 5C, qPCR). In all cases, signals of the ΔUL50 virus remained significantly lower than those of the parental WT, but nevertheless showed at least a slight increase of genomic loads after 4 d p.i. (Figure 5C). Thus, the ddPCR and qPCR data further illustrated a finding described already earlier [19]. The ΔUL50 rescue virions, even in the absence of pUL50-complementing cells, are not fully replication incompetent, but show some residual capacity of transient genome replication over a period of two to three viral replication cycles.

**Figure 6:**
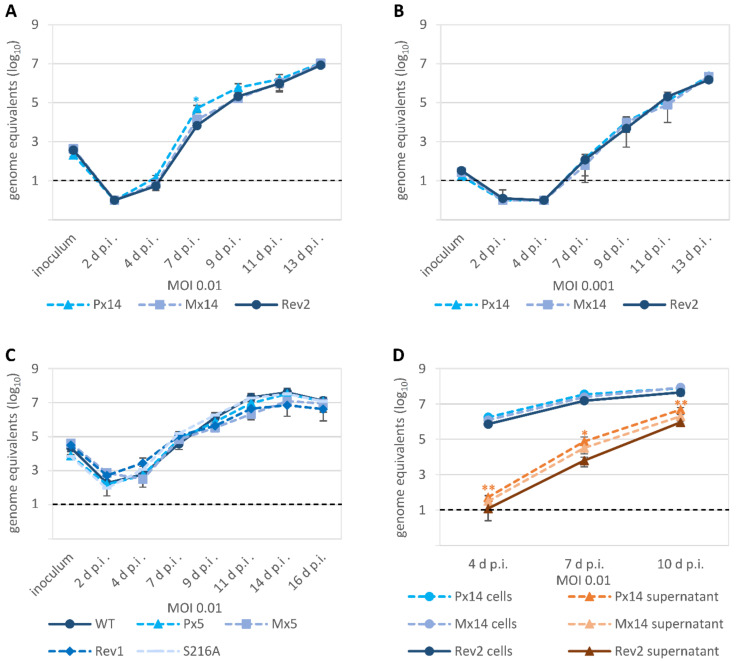
HCMV replication kinetics of the pUL50 phosphosite mutants. HFFs were infected with parental HCMV AD169 (WT) or recombinant HCMV phosphosite mutants P×14, M×14, Rev2, P×5, M×5, Rev1 or S216A at a MOI of 0.01 or 0.001 as indicated. Viral supernatants (**A**–**D**) or cells (**D**) were harvested at the indicated time points and viral genome equivalents were determined by IE1-specific qPCR. Each infection was performed at least in triplicates; mean values and standard deviations are shown. The significance is calculated relating to Rev2 or WT (solid lines). The standard containing 10 HCMV DNA copies reached the cycle threshold at cycle 38 and was therefore defined as the limit of detection for qPCR, as shown by the black dashed line. *, *p* ≤ 0.05; **, *p* ≤ 0.01.

All the ORF-UL50 phosphosite mutants showed almost WT-like replication kinetics and efficiency, indistinguishable from the control virus qPCR curves, so that no statistically significant impairment of progeny virus production was determined (Figure 6). This was true for both the P×5 and M×5 pair as well as the P×14 and M×14 pair (Figure 6A–C), for MOIs of 0.01 and 0.001 (reproduced by ddPCR with very similar results; data not shown), and for the use of supernatants parallel to cellular lysates as a PCR template (Figure 6D). Thus, the combined findings for the viral phosphosite replacement mutants do not suggest phenotypic alteration of viral replication kinetics.

### 3.5. Electron Microscopic Analysis of HFFs Infected with the Recombinant HCMVs

**Figure 7:**
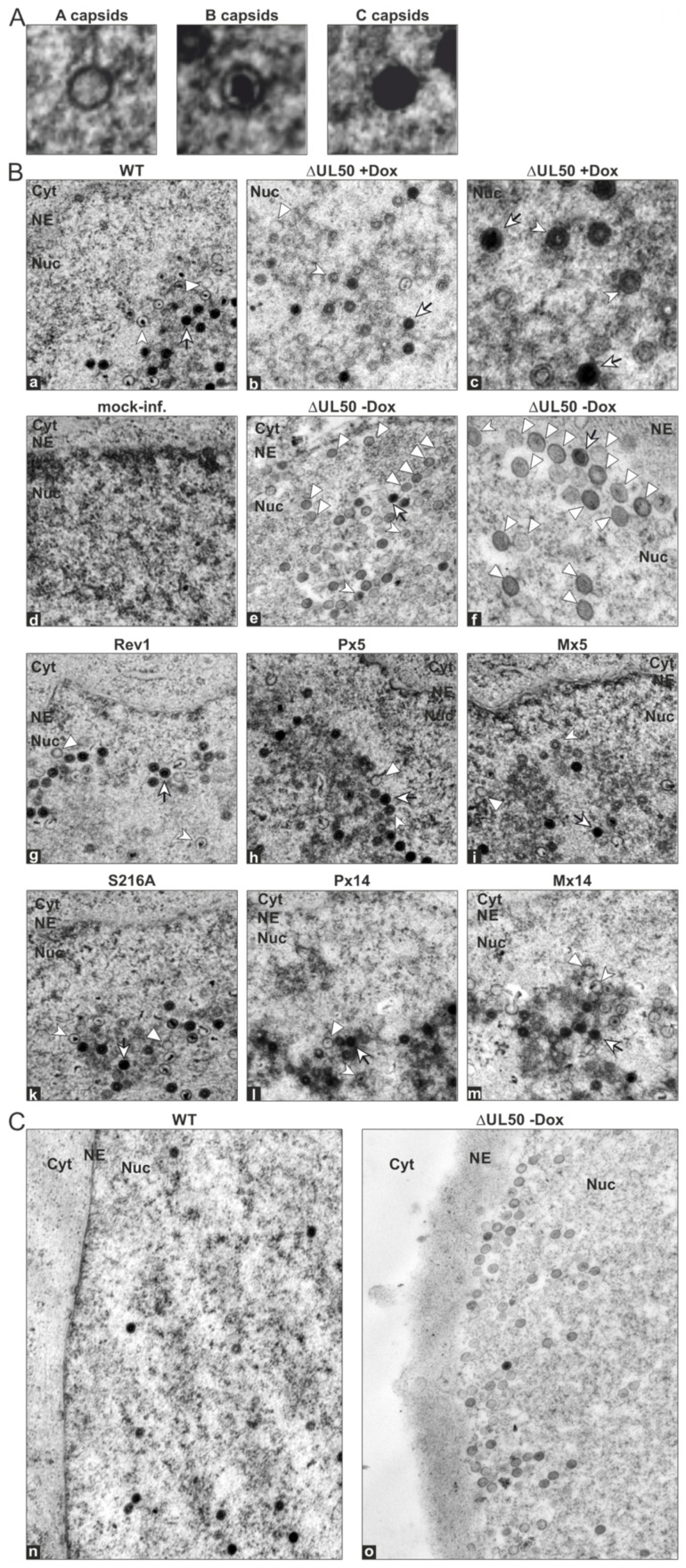
Electron microscopic analysis of viral nuclear capsid formation. HFFs were infected with parental WT, mutant or revertant HCMVs as indicated at a MOI adapted to maintain cell viability for harvesting and fixation at 4–7 d p.i., to be subjected to sectioning and negative staining in TEM. (**A**) Representatives of A-, B- and C-capsids. (**B**) Representative nuclei of infected cells at a 16,700-fold or 33,400-fold (c,f) magnification. The distribution of A- (white triangle), B- (white arrowhead) and C-capsids (white arrowhead with black tail) is depicted. NE, nuclear envelope; Cyt, cytoplasm; Nuc; nucleus. (**C**) Accumulation of capsids of HCMV ΔUL50 in close proximity to the nuclear envelope in contrast to a more homogeneous distribution of the WT (25,050-fold magnification).

**Figure 8:**
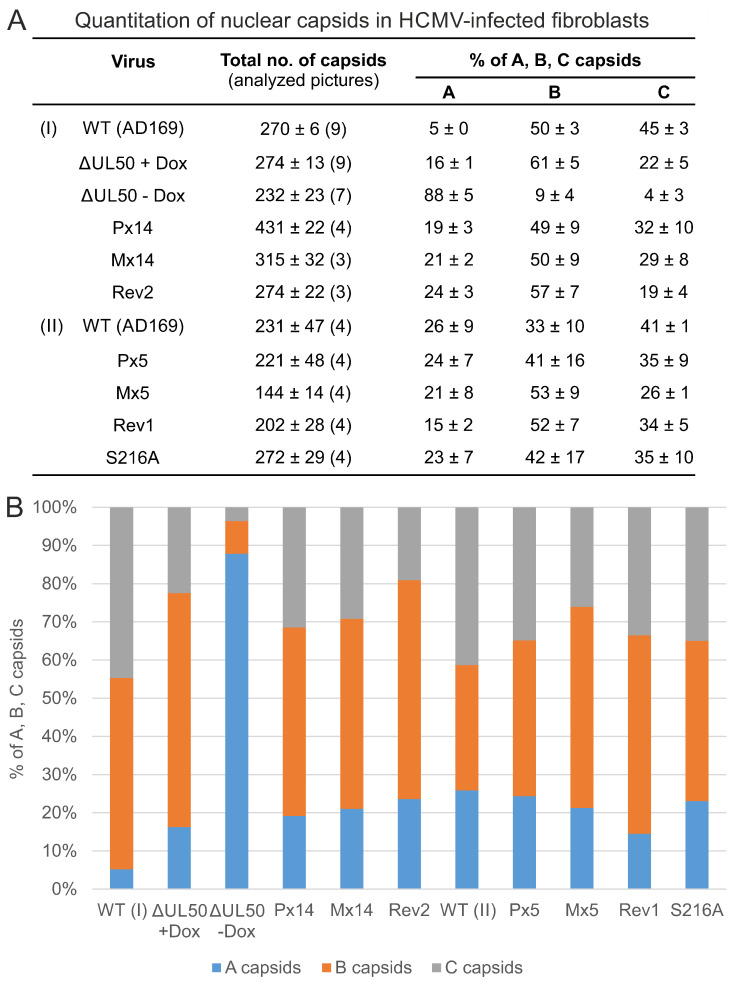
Quantitative evaluation of intranuclear capsid types. For the details of infection (4–7 d.p.i.) and sample preparation see Figure 7. (**A**) TEM counting was performed individually by researchers in duplicate (*n* = 4–8), using up to nine nuclear sections for each virus. Mean values ± SD of A, B and C capsids are given, as set in percentage to the total number of capsids counted. (**B**) Diagram representation of the mean values given above.

In order to assess the phenotype of viral nuclear capsid formation for WT, mutant and revertant HCMVs, transmission electron microscopy (TEM) analysis was performed (Figure 7 and Figure S6). Ultrathin sections of HCMV-infected HFFs were stained with uranyl acetate and lead citrate to visualize the nuclear types A, B and C of viral capsids (Figure 7A). Analysis was performed in two experimental settings (I, II) and a quantitation of capsid numbers was conducted by counting several nuclear sections for each virus (Figure 8A,B). In the majority, intermediate type B capsids were detected for WT, revertants as well as all phosphosite mutants (ranging between 33 ± 10% and 61 ± 5% for the individual viruses). Lower percentages were obtained for immature type A capsids (5 ± 0% to 26 ± 9%) and mature type C capsids (19 ± 4 to 45 ± 3%). Some degree of variation of percentages was noted between the two experimental settings, as expressed by the values of WT (I), WT (II), Rev1 and Rev2. No clear phenotypical alteration was found for any of the phosphosite mutants P×5, M×5, S216A, P×14 or M×14, neither in quantitative nor in qualitative terms (Figure 7, Figure 8 and Figure S6). Strikingly, however, the deletion mutant ΔUL50 showed a profoundly different pattern of capsids than WT. While the conditions of pUL50 complementation (+Dox) yielded capsid numbers for ΔUL50 in the range of other viruses, conditions of lack of pUL50 (−Dox) showed a drastic change in two aspects: first, the ΔUL50 mutant was characterized by a strong accumulation and entrapment of capsids at the nucleoplasmic proximity of the nuclear envelope (Figure 7C, NE); second, the quantitation revealed a strong abundance of immature A type capsids (Figure 8A,B, 88 ± 5%) and a drastic reduction of both B and C types (9 ± 4% and 4 ± 3%, respectively). This finding clearly confirmed the replicative defect of the ΔUL50 mutant, i.e., a lack of efficient nuclear capsid egress. The defect could be relieved by pUL50 complementation in +Dox cells. Obviously, the entrapment of intranuclear A and B capsids, aligned along the NE, was linked to an inefficiency of capsid maturation. No similar defect was observed for any of the phosphosite mutants (Figure 7 and Figure 8). Referring to the report by Krosky et al. [36], in which an EM-based quantitation of nuclear versus cytoplasmic capsids was described, we were not able to perform a similar quantitative assessment due to the very low number of detectable capsids in the cytoplasm of all samples (Figure S6). Combined our data strongly supported the conclusion that HCMV nuclear egress is massively hampered by ORF-UL50 deletion, but is poorly affected by the pUL50 phosphosite mutations analyzed in this study.

## 4. Discussion

In the present study we focused on the investigation of newly generated recombinant HCMVs carrying ORF-UL50 deletion or pUL50 phosphosite mutations. Their putative phenotypical changes in nuclear egress should be addressed and the rationale behind this, i.e., the question whether pUL50 phosphosite mutations would markedly alter viral replication efficiencies, originated from our previous proteomics-based identification of 14 distinct detectable phosphosites. The main findings are the following: (i) only the ORF-UL50 deletion rescue virus showed a block of genome synthesis at late infection periods (viral replication kinetics determined by qPCR/ddPCR), whereas the phosphosite mutants displayed largely unchanged kinetics, (ii) neither ORF-UL50 deletion nor phosphosite replacements led to drastic changes in viral protein expression patterns, (iii) confocal imaging of NEC colocalization and CoIP-based analysis of NEC interaction revealed three distinct protein examples (pUL53, pUL44, MCP) of differences in intracellular localization compared to WT for ORF-UL50 deletion, but not for phosphosite mutants, and (iv) TEM analysis revealed a strong abundance and accumulation of immature A-capsids for ΔUL50 in contrast to WT and pUL50 phosphosite mutants. On this basis, the current data underline the strong phenotypical changes of the ΔUL50 virus, whereas viruses carrying multiple pUL50 phosphosite mutations were found largely unaltered.

Generally, it is widely accepted knowledge that nuclear egress is determined by the functionality of herpesviral NEC proteins and represents an essential and rate-limiting step of viral replication efficiency (reviewed by [1,3,4,5,37,38,39,40,41]). A number of molecular characteristics have been identified for herpesviral core NEC proteins, among which both identical features and differences could be pointed out [1,5,9,10,42]. Especially for the example of HCMV pUL50-pUL53, at least three important functions have been experimentally described or postulated so far, namely the recruitment of NEC-associated effectors, the reorganization of the nuclear envelope and the NEC-docking and transition of nuclear capsids. Hereby, the role of regulatory phosphorylation of NEC-associated proteins moved more and more into the focus of our interest [43]. It should be stressed that most, if not all, of the HCMV core NEC and NEC-associated proteins are either subject to massive phosphorylation or represent NEC-/lamina-phosphorylating protein kinases themselves. A previous report described a replacement of S216A in pUL50 together with S19A in pUL53 resulting in the loss of efficient NEC rim location and viral replication [7]. Although this study showed some methodological differences compared to our data (e.g., different cell types used for transient transfection or HCMV infection, respectively, in addition pUL50/pUL53 double-mutant instead of pUL50 single-mutant), divergent results could not be explained at this stage. In order to address the apparent discrepancy, we reconstituted five BAC-derived HCMVs harboring various phosphosite patterns of pUL50. Confirming our initial results, we could not identify a functional impact of the pUL50 phosphosites under conditions of HCMV multiround replication in cultured primary fibroblasts. Although three parameters were intensively studied for these mutants, i.e., viral replication kinetics and protein production, NEC protein association and nuclear lamin phosphorylation and viral nuclear capsid egress (TEM analysis), no functional impairment during any stage of the viral replication cycle could be detected. Thus, we conclude that although pUL50 is heavily phosphorylated by viral and cellular kinases, our findings so far cannot substantiate the importance of phosphorylation at the analyzed 14 phosphosites in pUL50 for NEC functionality [7,8,23,44].

Combined, the findings suggest that alternative pUL50 phosphorylation was either not detectable or phosphorylation may generally not be functionally essential. This situation may prompt to speculate about the importance of additional minor phosphosites in pUL50 or, even more likely, on the basis of current data, in pUL53 [7,8]. The observations published so far strongly suggest that NEC is regulated by phosphorylation [1], even though phosphorylation of pUL50 itself had no effect on viral replication as described in this report. As a commonly accepted feature, the main regulatory protein kinase of the HCMV-specific NEC is the viral kinase pUL97. Its main NEC-associated viral and cellular phosphorylated substrates have been identified as the pUL50-pUL53 complex, pUL97 autophosphorylation, pp65 with unknown NEC impact, the multiligand-binding bridging factor p32/gC1qR, emerin and nuclear lamins A/C [45]. Additional cellular kinases, such as CDK1, can contribute to the phosphorylation of the core NEC and NEC-associated proteins [8,23,25,44]. To date, however, with the exception of lamin A/C phosphorylation, the regulatory impact of NEC-specific phosphorylation activities has not been clarified.

Considering this aspect of functional relevance of phosphosites beyond NEC regulation, multiple events of protein phosphorylation during human herpesviral and non-herpesviral infections were described for a variety of viruses, some of these with or without functional importance [46,47,48,49,50]. Radestock and colleagues demonstrated that the predominant HIV-1 phosphoprotein 6 (p6) comprised several phosphosites, but their substitution had no effect on p6 functions including virus release [48]. Regarding the functional relevance of multiple-site phosphorylation of nuclear proteins more related to our study, examples have been described for two human and animal α-herpesviruses. The bovine herpesvirus 1 (BoHV-1) nuclear protein VP8 was found to be phosphorylated by both cellular and viral kinases at various sites, thereby promoting viral replication [50]. With regard to core NEC proteins, the phosphorylation of six N-terminal serine residues of HSV-1 pUL31 by viral kinase pUS3 was described. These sites were characterized as indispensable for capsid release from the nucleus and thus their functional relevance during HSV-1 infection was postulated [51]. More recent data implied that some of these residues within the extreme N-terminus of pUL31 might be crucial for nuclear egress. Substitutions of four phosphorylated residues resulted in capsid accumulations adjacent to the inner nuclear membrane, whereas single substitutions had no detectable effect [52].

Intriguingly, the major phosphosites are not positionally conserved throughout the pUL50 protein family in α-, β- and γ-herpesviruses. Alignments revealed that HCMV major phosphosites seem to be exclusively present in pUL50 but not in other herpesviral homologs [14]. However, our sequence analysis indicated some partial homologies; i.e., S216 of HCMV pUL50 was found in human herpesvirus 6 and 7 (HHV-6 and HHV-7; S216) homologs but not in MCMV. Some additional alignment patterns such as the proline-directed kinase motif, comprising a serine residue with a subsequent proline (Ser-Pro), have been identified in VZV (S209) and PrV (S194) homologs. These short sequence sections point to a possible substitutional relevance of alternative serine residues within the respective pUL50 homologs. Moreover, the relevant phosphosites identified so far are predominantly located in non-globular regions of pUL50; thus, positions of putative phosphosites within pUL50 homologs might differ and are not easily detectable by alignment approaches.

Combined, this report strongly suggests the conserved functional importance of the cytomegalovirus core NEC proteins. In particular, our data underline the central, albeit not fully essential, role of pUL50 as a multi-interacting determinant of the HCMV core NEC, at least considering the efficiency of multiple rounds of viral lytic replication. The study suggests a refined model of pUL50 functionality, in which the regulation of viral replication, specifically the nuclear egress, seems to allow some compensatory measures to balance alterations in the expression and phosphorylation levels of pUL50. It has not escaped our notice that pUL50 is not only present at low copy numbers in infectious virions [53], but also comprises additional, NEC-independent activities [54,55]. Thus, more detailed studies on the virion-associated state of pUL50 may help to understand this multifaceted regulatory aspect in the near future.

## Data Availability

Not Applicable.

## Supplementary Materials

The following are available online at https://www.mdpi.com/1999-4915/13/2/165/s1. Figure S1, Schematic representation of the generation of recombinant HCMVs; Figure S2, CoIP-based interaction analysis of pUL50 phophosite mutants; Figure S3, Confocal imaging analysis of the localization of pUL50 and pUL53 of transfected phosphosite mutants; Figure S4, Confocal imaging analysis of the localization of cellular proteins produced by HCMV ΔUL50 or pUL50 phosphosite mutations; Figure S5, HCMV replication kinetics of the ORF-UL50 deletion mutant; Figure S6, Electron microscopic investigation of recombinant viruses; Figure S7: Sequence alignments of ORFs UL50, UL53 and UL97 of reconstituted viruses.
