# Supplementary material for: Phenotypical Characterization of the Nuclear Egress of Recombinant Cytomegaloviruses Reveals Defective Replication upon ORF-UL50 Deletion but Not pUL50 Phosphosite Mutation
Source: Viruses. 2021 Jan 22;13(2):165. doi: 10.3390/v13020165 (PMC7911381; doi:10.3390/v13020165)
Supplement: Supplementary file 1 [file viruses-13-00165-s001.pdf]

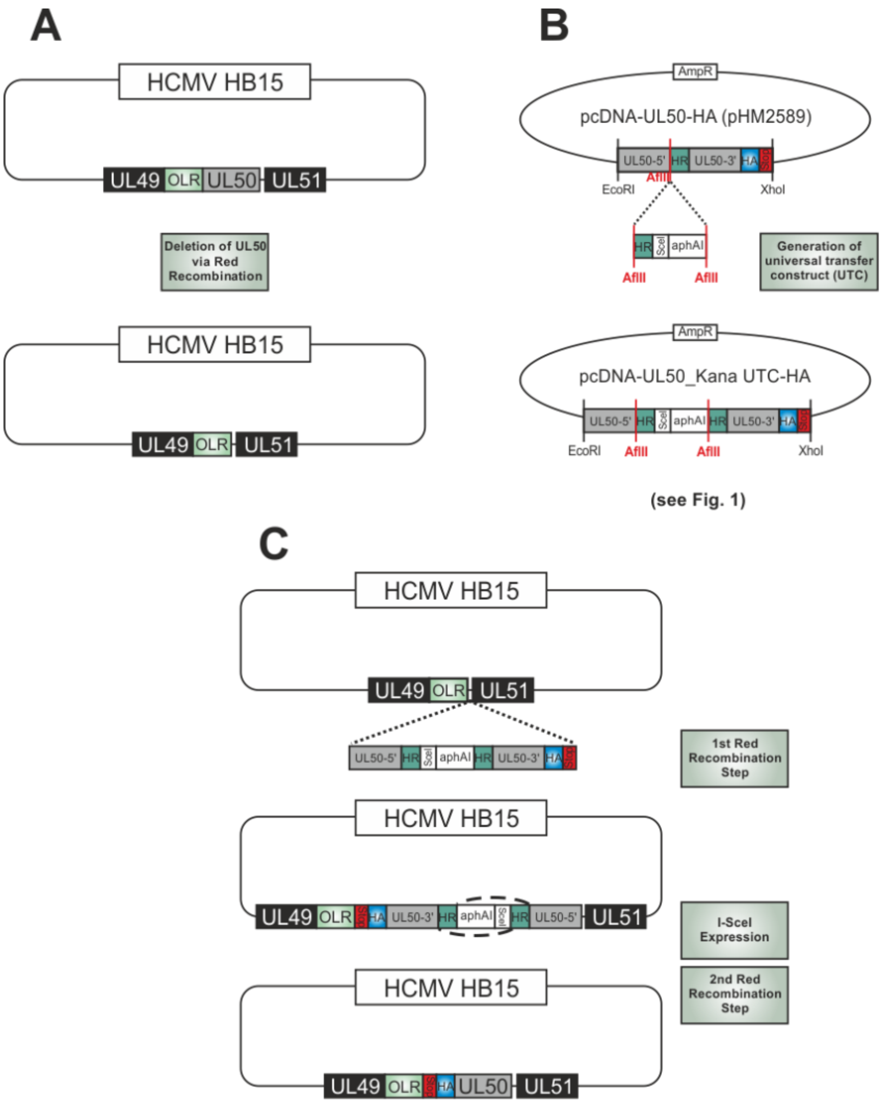

Figure S1. Schematic representation of the generation of recombinant HCMVs. **(A)** pUL50 was partially deleted from the HB15 BACmid using Red recombination without affecting the pUL49/pUL50 overlapping region (OLR, highlighted in light green). **(B)** Generation of UTCs, here depicted for wild-type pUL50-HA. Constructs containing homologous regions (HR) important for the second step of Red recombination, an I-SceI restriction site and a kanamycin cassette (aphAI) were inserted into pcDNA3.1 plasmids harboring pUL50-HA, pUL50(S216A)-HA, pUL50(Px5)-HA, pUL50(Mx5)-HA, pUL50, pUL50(Px14), pUL50(Mx14) via an AflII restriction site. The HA-tag is highlighted in blue and the stop codon in red. **(C)** Exemplary insertion of wild-type ORF UL50-HA (Rev1) Kana into UL50-deleted HB15 HB15/AD169ΔUL50. Prior to transformation, homologous regions for insertion into the BACmid were amplified in the respective UTCs and subsequently transformed into bacteria containing HB15/AD169ΔUL50 [19]. After I-SceI induction, the second step of Red recombination occurred at homologous regions (HRs, dashed lines) and the kanamycin cassette was deleted from the backbone resulting in the WT pUL50-HA (Rev1) construct.

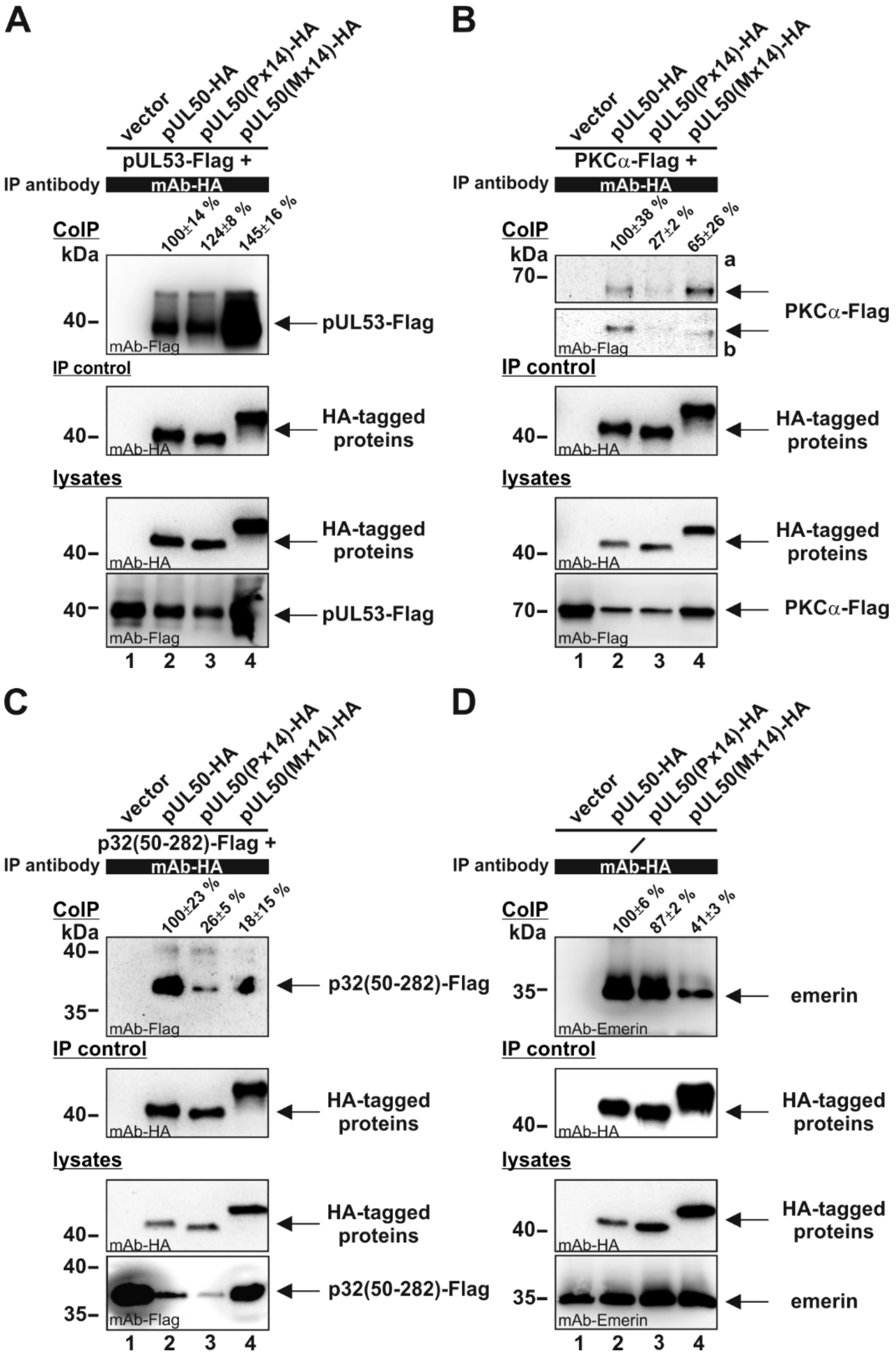

Figure S2. CoIP-based interaction analysis of pUL50 phophosite mutants. 293T cells were transiently transfected with expression plasmids coding for HA-tagged pUL50, pUL50(Px14) or pUL50(Mx14) and Flag-tagged pUL53, PKC*α* or p32(50-282). At three d p.t., cells were lysed and HA-tagged proteins were immunoprecipitated using mAb-HA. Lysate controls taken prior to the IP and CoIP samples were subjected to standard Wb analysis using tag-specific antibodies as indicated. Signal intensities of pUL50 interaction partners pUL53 **(A)**, PKC*α* **(B)**, p32(50-282) **(C)** and emerin **(D)** were quantified using Aida Image Analyzer v.4.23.

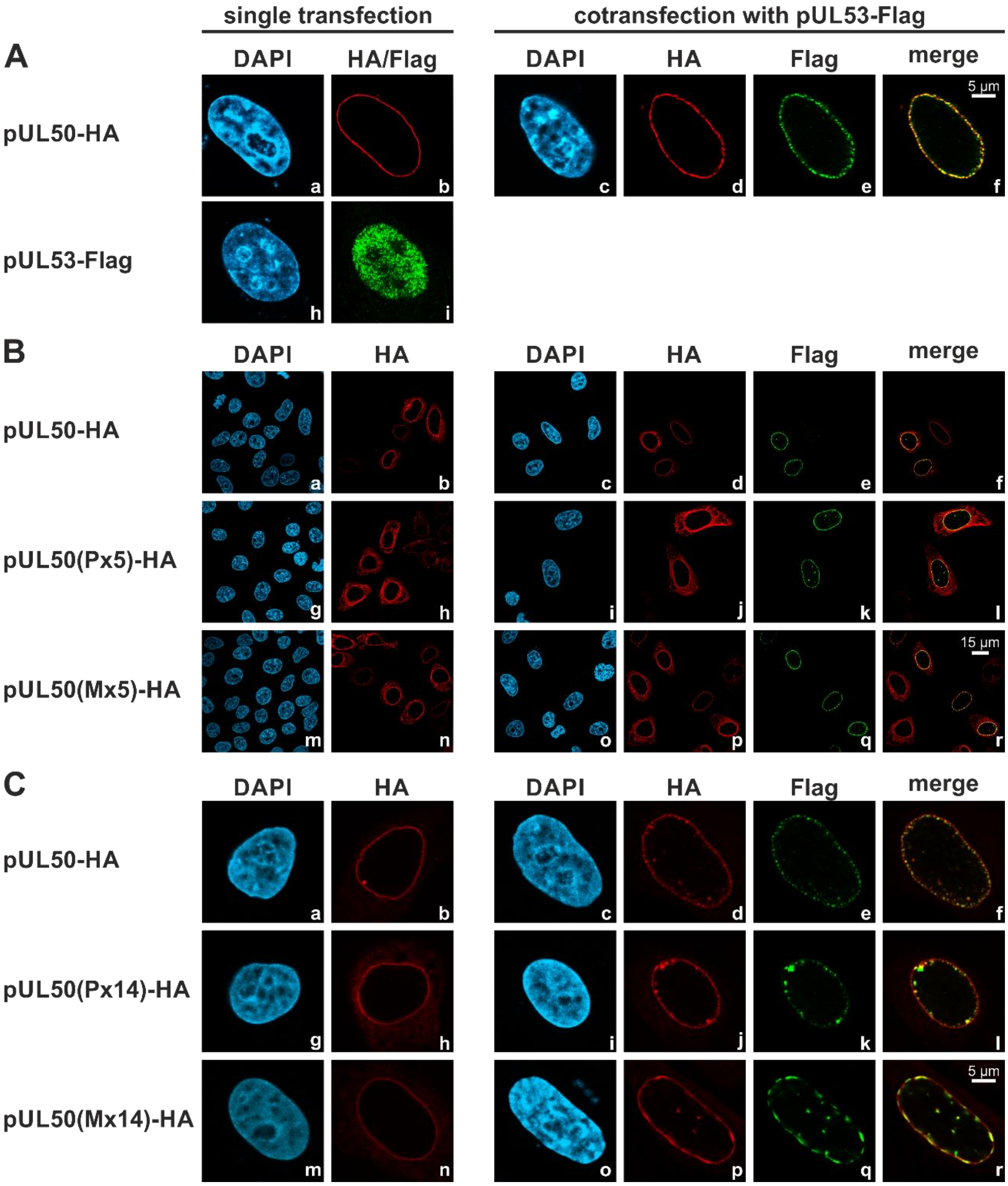

Figure S3. Confocal imaging analysis of the localization of pUL50 and pUL53 of transfected phosphosite mutants. HeLa cells were transiently transfected with constructs coding for pUL50-HA, pUL53-Flag, pUL50(Px5)-HA, pUL50(Mx5)-HA, pUL50(Px14)-HA, pUL50(Mx14)-HA (left panel) or cotransfected with pUL53-Flag (right panel). Two d p.t., cells were fixed and used for immunostaining with tag-specific antibodies analyzed by confocal imaging. **(A)** WT constructs for pUL50 and pUL53. **(B)** Phosphosite mutants with five exchanged phosphosites. **(C)** Phosphosite mutants with 14 exchanged phosphosites.

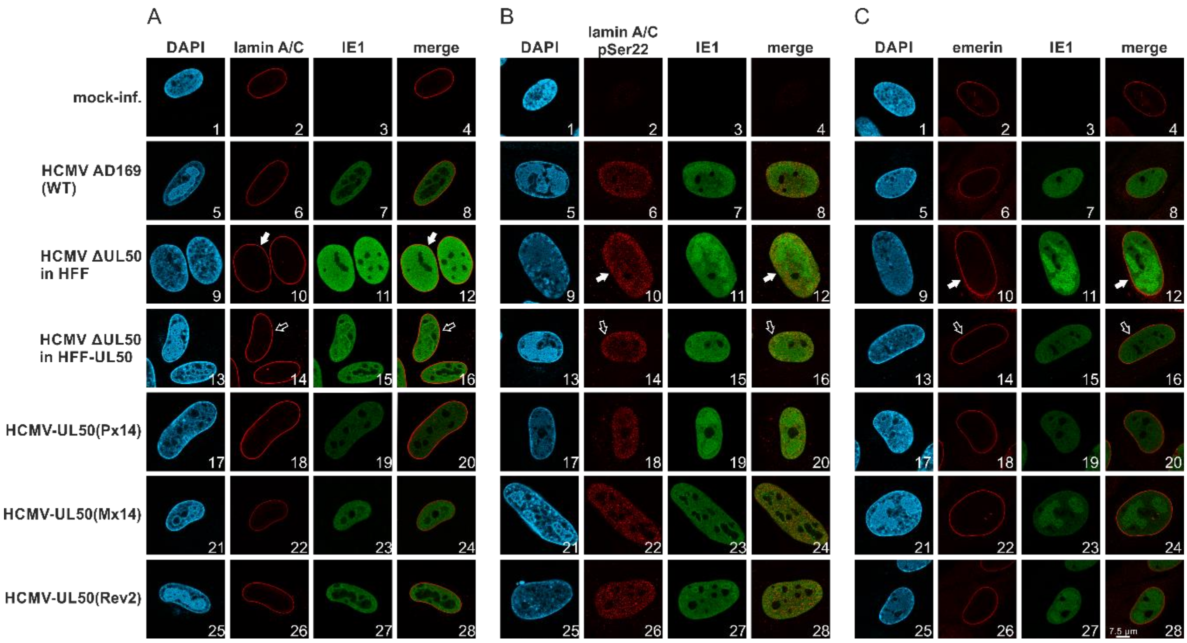

Figure S4. Confocal imaging analysis of the localization of cellular proteins produced by HCMV ΔUL50 or pUL50 phosphosite mutations. Normal HFFs or pUL50-complementing cells (HFF-UL50 +Dox) were used for infection with the recombinant HCMVs at a MOI of 0.05 and harvested at 7 d p.i.. Immunofluorescence staining was performed with antibodies against the indicated proteins and representative panels of confocal imaging are given (see scale bar in panel C, picture 28). **(A-C)** Cellular proteins lamin A/C, lamin A/C pSer22 and emerin, showing no alteration in localization patterns.

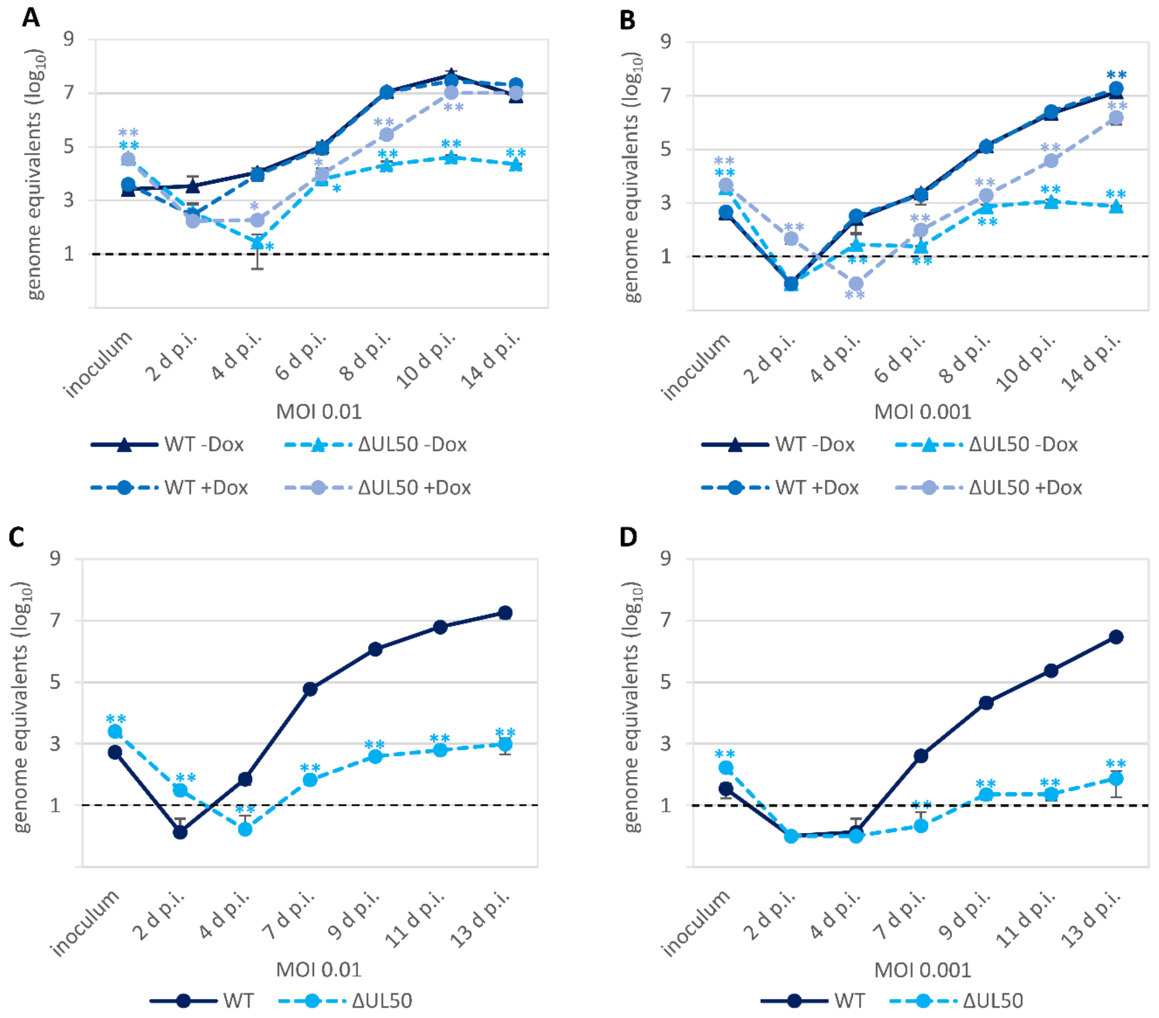

Figure S5. HCMV replication kinetics of the ORF-UL50 deletion mutant. HFF-UL50 cells **(A, B)** or HFFs **(C, D)** were infected with parental HCMV AD169 (WT) or recombinant HCMV ΔUL50 at a MOI of 0.01 **(A, C)** or 0.001 **(B, D)**. Viral supernatants were harvested at the indicated time point and viral genome equivalents released into the supernatant were determined by IE1-specific quantitative real-time PCR. Each infection was performed in triplicate; mean values and standard deviations are shown. The significance is calculated relating to WT (solid lines). The black dashed line shows the limit of detection. *, $p \leq 0.05$; **, $p \leq 0.01$

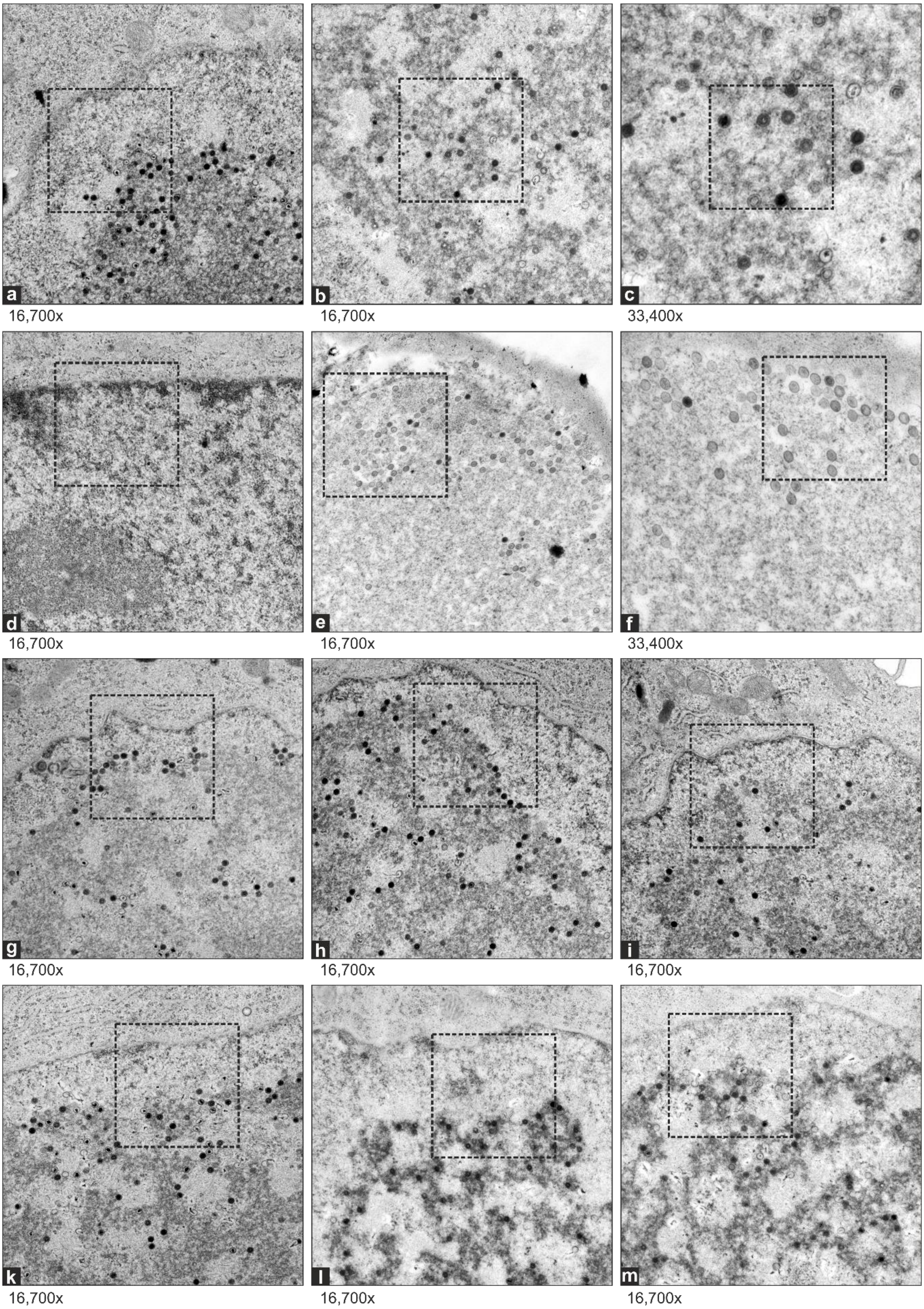

Figure S6. Electron microscopic investigation of recombinant viruses. HFFs or HFF-UL50 (b,c) remained mock-infected (d) or were infected with WT (a), ΔUL50 +Dox (b,c), ΔUL50 –Dox (e,f), Rev1 (g), Px5 (h), Mx5 (i), S216A (k), Px14 (l) or Mx14 (m) and were fixed at 4-7 d p.i. Samples were analyzed by TEM; magnification as indicated. Dashed boxes indicate enlarged pictures shown in Figure 7.

A

UL50 Sequences

```
WT          MEMNKVLHQDLVQATRRILKLGPSELRVTDAGLICKNPNYSVCDAMLKTDTVYCVEYLLS 60
ΔUL50       ------------------------------------------------------------
Px5         MEMNKVLHQDLVQATRRILKLGPSELRVTDAGLICKNPNYSVCDAMLKTDTVYCVEYLLS
Mx5         MEMNKVLHQDLVQATRRILKLGPSELRVTDAGLICKNPNYSVCDAMLKTDTVYCVEYLLS
Rev1        MEMNKVLHQDLVQATRRILKLGPSELRVTDAGLICKNPNYSVCDAMLKTDTVYCVEYLLS
Px14        MEMNKVLHQDLVQATRRILKLGPSELRVTDAGLICKNPNYSVCDAMLKTDTVYCVEYLLS
Mx14        MEMNKVLHQDLVQATRRILKLGPSELRVTDAGLICKNPNYSVCDAMLKTDTVYCVEYLLS
Rev2        MEMNKVLHQDLVQATRRILKLGPSELRVTDAGLICKNPNYSVCDAMLKTDTVYCVEYLLS

WT          YWESRTDHVPCFIFKNTGCAVSLCCFVRAPVKLVSPARHVGEFNVLKVNESLIVTLKDIE 120
ΔUL50       ------------------------------------------------------------
Px5         YWESRTDHVPCFIFKNTGCAVSLCCFVRAPVKLVSPARHVGEFNVLKVNESLIVTLKDIE
Mx5         YWESRTDHVPCFIFKNTGCAVSLCCFVRAPVKLVSPARHVGEFNVLKVNESLIVTLKDIE
Rev1        YWESRTDHVPCFIFKNTGCAVSLCCFVRAPVKLVSPARHVGEFNVLKVNESLIVTLKDIE
Px14        YWESRTDHVPCFIFKNTGCAVSLCCFVRAPVKLVAPARHVGEFNVLKVNESLIVTLKDIE
Mx14        YWESRTDHVPCFIFKNTGCAVSLCCFVRAPVKLVEPARHVGEFNVLKVNESLIVTLKDIE
Rev2        YWESRTDHVPCFIFKNTGCAVSLCCFVRAPVKLVSPARHVGEFNVLKVNESLIVTLKDIE

WT          EIKPSAYGVLTKCVVRKSNSASVFNIELIAFGPENEGEYENLLRELYAKKAASTSLAVRN 180
ΔUL50       ------------------------------------------------------------
Px5         EIKPSAYGVLTKCVVRKSNSASVFNIELIAFGPENEGEYENLLRELYAKKAASTSLAVRN
Mx5         EIKPSAYGVLTKCVVRKSNSASVFNIELIAFGPENEGEYENLLRELYAKKAASTSLAVRN
Rev1        EIKPSAYGVLTKCVVRKSNSASVFNIELIAFGPENEGEYENLLRELYAKKAASTSLAVRN
Px14        EIKPSAYGVLTKCVVRKSNSASVFNIELIAFGPENEGEYENLLRELYAKKAASASLAVRN
Mx14        EIKPSAYGVLTKCVVRKSNSASVFNIELIAFGPENEGEYENLLRELYAKKAASESLAVRN
Rev2        EIKPSAYGVLTKCVVRKSNSASVFNIELIAFGPENEGEYENLLRELYAKKAASTSLAVRN

WT          HVTVSSHSGSGPSLWRARMSAALTRTAGKRSSRTASPPPPPRHPSCSPTMVAAGGAAAGP 239
ΔUL50       ------------------------------------------------------------
Px5         HVTVSSHAGSGPSLWRARMSAALTRTAGKRSSRAAAPPPPPRHPSCSPTMVAAGGAAAGP
Mx5         HVTVSSHEGSGPSLWRARMSAALTRTAGKRSSREAEPPPPPRHPSCSPTMVAAGGAAAGP
Rev1        HVTVSSHSGSGPSLWRARMSAALTRTAGKRSSRTASPPPPPRHPSCSPTMVAAGGAAAGP
Px14        HVAVSSHAGSGPSLWRARMSAALTRTAGKRSSRAAAPPPPPRHPACAPAMVAAGGAAAGP
Mx14        HVEVSSHEGSGPSLWRARMSAALTRTAGKRSSREAEPPPPPRHPECEPEMVAAGGAAAGP
Rev2        HVTVSSHSGSGPSLWRARMSAALTRTAGKRSSRTASPPPPPRHPSCSPTMVAAGGAAAGP
```

```
WT         RPPPPPMAAGSWRLCRCEACMGRCGCASEGDADEEEEELLALAGEGKAAAAAAGQDVGGS 299
ΔUL50      ------------------------------------------------------------
Px5        RPPPPPMAAGSWRLCRCEACMGRCGCASEGDADEEEEELLALAGEGKAAAAAAGQDVGGA
Mx5        RPPPPPMAAGSWRLCRCEACMGRCGCASEGDADEEEEELLALAGEGKAAAAAAGQDVGGE
Rev1       RPPPPPMAAGSWRLCRCEACMGRCGCASEGDADEEEEELLALAGEGKAAAAAAGQDVGGS
Px14       RPPPPPMAAGAWRLCRCEACMGRCGCAAEGDADEEEEELLALAGEGKAAAAAAGQDVGGA
Mx14       RPPPPPMAAGEWRLCRCEACMGRCGCAEEGDADEEEEELLALAGEGKAAAAAAGQDVGGE
Rev2       RPPPPPMAAGSWRLCRCEACMGRCGCASEGDADEEEEELLALAGEGKAAAAAAGQDVGGS

WT         ARRPLEEHVSRRRGVSTHHRHPPSPPCAPSLERTGYRWAPSSWWRARSGPSRPQSGPWLP 359
ΔUL50      ------------------------------------------------------------
Px5        ARRPLEEHVSRRRGVSTHHRHPPAPPCAPSLERTGYRWAPSSWWRARSGPSRPQSGPWLP
Mx5        ARRPLEEHVSRRRGVSTHHRHPPEPPCAPSLERTGYRWAPSSWWRARSGPSRPQSGPWLP
Rev1       ARRPLEEHVSRRRGVSTHHRHPPSPPCAPSLERTGYRWAPSSWWRARSGPSRPQSGPWLP
Px14       ARRPLEEHVSRRRGVSTHHRHPPAPPCAPALERTGYRWAPSSWWRARSGPSRPQSGPWLP
Mx14       ARRPLEEHVSRRRGVSTHHRHPPEPPCAPELERTGYRWAPSSWWRARSGPSRPQSGPWLP
Rev2       ARRPLEEHVSRRRGVSTHHRHPPSPPCAPSLERTGYRWAPSSWWRARSGPSRPQSGPWLP

WT         ARFATLGPLVLALLLVLALLWRGHGQSSSPTRSAHRD---------       396
ΔUL50      ------------------------GQSSSPTRSAHRD--------
Px5        ARFATLGPLVLALLLVLALLWRGHGQSSSPTRSAHRDYPYDVPDYA
Mx5        ARFATLGPLVLALLLVLALLWRGHGQSSSPTRSAHRDYPYDVPDYA
Rev1       ARFATLGPLVLALLLVLALLWRGHGQSSSPTRSAHRDYPYDVPDYA
Px14       ARFATLGPLVLALLLVLALLWRGHGQSSSPTRSAHRD---------
Mx14       ARFATLGPLVLALLLVLALLWRGHGQSSSPTRSAHRD---------
Rev2       ARFATLGPLVLALLLVLALLWRGHGQSSSPTRSAHRD---------
                                   *************
```

B

UL53 Sequences

```
WT         MSSVSGVRTPRERRSALRSLLRKRRQRELASKVASTVNGATSANNHGEPPSPADARPRLT 60
ΔUL50      MSSVSGVRTPRERRSALRSLLRKRRQRELASKVASTVNGATSANNHGEPPSPADARPRLT
Px5        MSSVSGVRTPRERRSALRSLLRKRRQRELASKVASTVNGATSANNHGEPPSPADARPRLT
Mx5        MSSVSGVRTPRERRSALRSLLRKRRQRELASKVASTVNGATSANNHGEPPSPADARPRLT
Rev1       MSSVSGVRTPRERRSALRSLLRKRRQRELASKVASTVNGATSANNHGEPPSPADARPRLT
Px14       MSSVSGVRTPRERRSALRSLLRKRRQRELASKVASTVNGATSANNHGEPPSPADARPRLT
Mx14       MSSVSGVRTPRERRSALRSLLRKRRQRELASKVASTVNGATSANNHGEPPSPADARPRLT
Rev2       MSSVSGVRTPRERRSALRSLLRKRRQRELASKVASTVNGATSANNHGEPPSPADARPRLT
           ************************************************************

WT         LHDLHDIFREHPELELKYLNMMKMAITGKESICLPFNFHSHRQHTCLDISPYGNEQVSRI 120
ΔUL50      LHDLHDIFREHPELELKYLNMMKMAITGKESICLPFNFHSHRQHTCLDISPYGNEQVSRI
Px5        LHDLHDIFREHPELELKYLNMMKMAITGKESICLPFNFHSHRQHTCLDISPYGNEQVSRI
```

```
Mx5         LHDLHDIFREHPELELKYLNMMKMAITGKESICLPFNFHSHRQHTCLDISPYGNEQVSRI
Rev1        LHDLHDIFREHPELELKYLNMMKMAITGKESICLPFNFHSHRQHTCLDISPYGNEQVSRI
Px14        LHDLHDIFREHPELELKYLNMMKMAITGKESICLPFNFHSHRQHTCLDISPYGNEQVSRI
Mx14        LHDLHDIFREHPELELKYLNMMKMAITGKESICLPFNFHSHRQHTCLDISPYGNEQVSRI
Rev2        LHDLHDIFREHPELELKYLNMMKMAITGKESICLPFNFHSHRQHTCLDISPYGNEQVSRI
            ************************************************************

WT          ACTSCEDNRILPTASDAMVAFINQTSNIMKNRNFYYGFCKSSELLKLSTNQPPIFQIYYL 180
ΔUL50       ACTSCEDNRILPTASDAMVAFINQTSNIMKNRNFYYGFCKSSELLKLSTNQPPIFQIYYL
Px5         ACTSCEDNRILPTASDAMVAFINQTSNIMKNRNFYYGFCKSSELLKLSTNQPPIFQIYYL
Mx5         ACTSCEDNRILPTASDAMVAFINQTSNIMKNRNFYYGFCKSSELLKLSTNQPPIFQIYYL
Rev1        ACTSCEDNRILPTASDAMVAFINQTSNIMKNRNFYYGFCKSSELLKLSTNQPPIFQIYYL
Px14        ACTSCEDNRILPTASDAMVAFINQTSNIMKNRNFYYGFCKSSELLKLSTNQPPIFQIYYL
Mx14        ACTSCEDNRILPTASDAMVAFINQTSNIMKNRNFYYGFCKSSELLKLSTNQPPIFQIYYL
Rev2        ACTSCEDNRILPTASDAMVAFINQTSNIMKNRNFYYGFCKSSELLKLSTNQPPIFQIYYL
            ************************************************************

WT          LHAANHDIVPFMHAEDGRLHMHVIFENPDVHIPCDCITQMLTAAREDYSVTLNIVRDHVV 240
ΔUL50       LHAANHDIVPFMHAEDGRLHMHVIFENPDVHIPCDCITQMLTAAREDYSVTLNIVRDHVV
Px5         LHAANHDIVPFMHAEDGRLHMHVIFENPDVHIPCDCITQMLTAAREDYSVTLNIVRDHVV
Mx5         LHAANHDIVPFMHAEDGRLHMHVIFENPDVHIPCDCITQMLTAAREDYSVTLNIVRDHVV
Rev1        LHAANHDIVPFMHAEDGRLHMHVIFENPDVHIPCDCITQMLTAAREDYSVTLNIVRDHVV
Px14        LHAANHDIVPFMHAEDGRLHMHVIFENPDVHIPCDCITQMLTAAREDYSVTLNIVRDHVV
Mx14        LHAANHDIVPFMHAEDGRLHMHVIFENPDVHIPCDCITQMLTAAREDYSVTLNIVRDHVV
Rev2        LHAANHDIVPFMHAEDGRLHMHVIFENPDVHIPCDCITQMLTAAREDYSVTLNIVRDHVV
            ************************************************************

WT          ISVLCHAVSASSVKIDVTILQRKIDEMDIPNDVSESFERYKELIQELCQSSGNNLYEEAT 300
ΔUL50       ISVLCHAVSASSVKIDVTILQRKIDEMDIPNDVSESFERYKELIQELCQSSGNNLYEEAT
Px5         ISVLCHAVSASSVKIDVTILQRKIDEMDIPNDVSESFERYKELIQELCQSSGNNLYEEAT
Mx5         ISVLCHAVSASSVKIDVTILQRKIDEMDIPNDVSESFERYKELIQELCQSSGNNLYEEAT
Rev1        ISVLCHAVSASSVKIDVTILQRKIDEMDIPNDVSESFERYKELIQELCQSSGNNLYEEAT
Px14        ISVLCHAVSASSVKIDVTILQRKIDEMDIPNDVSESFERYKELIQELCQSSGNNLYEEAT
Mx14        ISVLCHAVSASSVKIDVTILQRKIDEMDIPNDVSESFERYKELIQELCQSSGNNLYEEAT
Rev2        ISVLCHAVSASSVKIDVTILQRKIDEMDIPNDVSESFERYKELIQELCQSSGNNLYEEAT
            ************************************************************
```

```
WT          SSYAIRSPLTASPLHVVSTNGCGPSSSSQSTPPHLHPPSQATQPHHYSHHQSQSQQHHHR 360
ΔUL50       SSYAIRSPLTASPLHVVSTNGCGPSSSSQSTPPHLHPPSQATQPHHYSHHQSQSQQHHHR
Px5         SSYAIRSPLTASPLHVVSTNGCGPSSSSQSTPPHLHPPSQATQPHHYSHHQSQSQQHHHR
Mx5         SSYAIRSPLTASPLHVVSTNGCGPSSSSQSTPPHLHPPSQATQPHHYSHHQSQSQQHHHR
Rev1        SSYAIRSPLTASPLHVVSTNGCGPSSSSQSTPPHLHPPSQATQPHHYSHHQSQSQQHHHR
Px14        SSYAIRSPLTASPLHVVSTNGCGPSSSSQSTPPHLHPPSQATQPHHYSHHQSQSQQHHHR
Mx14        SSYAIRSPLTASPLHVVSTNGCGPSSSSQSTPPHLHPPSQATQPHHYSHHQSQSQQHHHR
Rev2        SSYAIRSPLTASPLHVVSTNGCGPSSSSQSTPPHLHPPSQATQPHHYSHHQSQSQQHHHR
            ************************************************************

WT          PQSPPPPLFLNSIRAP      375
ΔUL50       PQSPPPPLFLNSIRAP
Px5         PQSPPPPLFLNSIRAP
Mx5         PQSPPPPLFLNSIRAP
Rev1        PQSPPPPLFLNSIRAP
Px14        PQSPPPPLFLNSIRAP
Mx14        PQSPPPPLFLNSIRAP
Rev2        PQSPPPPLFLNSIRAP
            ****************
```

C

UL97 Sequences

```
WT          MSSALRSRARSASLGTTTQGWDPPPLRRPSRARRRQWMREAAQAAAQAAVQAAQAAAAQV 60
ΔUL50       MSSALRSRARSASLGTTTQGWDPPPLRRPSRARRRQWMREAAQAAAQAAVQAAQAAAAQV
Px5         MSSALRSRARSASLGTTTQGWDPPPLRRPSRARRRQWMREAAQAAAQAAVQAAQAAAAQV
Mx5         MSSALRSRARSASLGTTTQGWDPPPLRRPSRARRRQWMREAAQAAAQAAVQAAQAAAAQV
Rev1        MSSALRSRARSASLGTTTQGWDPPPLRRPSRARRRQWMREAAQAAAQAAVQAAQAAAAQV
Px14        MSSALRSRARSASLGTTTQGWDPPPLRRPSRARRRQWMREAAQAAAQAAVQAAQAAAAQV
Mx14        MSSALRSRARSASLGTTTQGWDPPPLRRPSRARRRQWMREAAQAAAQAAVQAAQAAAAQV
Rev2        MSSALRSRARSASLGTTTQGWDPPPLRRPSRARRRQWMREAAQAAAQAAVQAAQAAAAQV
            ************************************************************

WT          AQAHVDENEVVDLMADEAGGGVTTLTTLSSVSTTTVLGHATFSACVRSDVMRDGEKEDAA 120
ΔUL50       AQAHVDENEVVDLMADEAGGGVTTLTTLSSVSTTTVLGHATFSACVRSDVMRDGEKEDAA
Px5         AQAHVDENEVVDLMADEAGGGVTTLTTLSSVSTTTVLGHATFSACVRSDVMRDGEKEDAA
Mx5         AQAHVDENEVVDLMADEAGGGVTTLTTLSSVSTTTVLGHATFSACVRSDVMRDGEKEDAA
Rev1        AQAHVDENEVVDLMADEAGGGVTTLTTLSSVSTTTVLGHATFSACVRSDVMRDGEKEDAA
Px14        AQAHVDENEVVDLMADEAGGGVTTLTTLSSVSTTTVLGHATFSACVRSDVMRDGEKEDAA
Mx14        AQAHVDENEVVDLMADEAGGGVTTLTTLSSVSTTTVLGHATFSACVRSDVMRDGEKEDAA
Rev2        AQAHVDENEVVDLMADEAGGGVTTLTTLSSVSTTTVLGHATFSACVRSDVMRDGEKEDAA
            ************************************************************
```

```
WT        SDKENLRRPVVPSTSSRGSAASGDGYHGLRCRETSAMWSFEYDRDGDVTSVRRALFTGGS 180
ΔUL50     SDKENLRRPVVPSTSSRGSAASGDGYHGLRCRETSAMWSFEYDRDGDVTSVRRALFTGGS
Px5       SDKENLRRPVVPSTSSRGSAAGGDGYHGLRCRETSAMWSFEYDRDGDVTSVRRALFTGGS
Mx5       SDKENLRRPVVPSTSSRGSAAGGDGYHGLRCRETSAMWSFEYDRDGDVTSVRRALFTGGS
Rev1      SDKENLRRPVVPSTSSRGSAAGGDGYHGLRCRETSAMWSFEYDRDGDVTSVRRALFTGGS
Px14      SDKENLRRPVVPSTSSRGSAASGDGYHGLRCRETSAMWSFEYDRDGDVTSVRRALFTGGS
Mx14      SDKENLRRPVVPSTSSRGSAASGDGYHGLRCRETSAMWSFEYDRDGDVTSVRRALFTGGS
Rev2      SDKENLRRPVVPSTSSRGSAASGDGYHGLRCRETSAMWSFEYDRDGDVTSVRRALFTGGS
          ********************* **************************************

WT        DPSDSVSGVRGGRKRPLRPPLVSLARTPLCRRRVGGVDAVLEENDVELRAESQDSAVASG 240
ΔUL50     DPSDSVSGVRGGRKRPLRPPLVSLARTPLCRRRVGGVDAVLEENDVELRAESQDSAVASG
Px5       DPSDSVSGVRGGRKRPLRPPLVSLARTPLCRRRVGGVDAVLEENDVELRAESQDSAVASG
Mx5       DPSDSVSGVRGGRKRPLRPPLVSLARTPLCRRRVGGVDAVLEENDVELRAESQDSAVASG
Rev1      DPSDSVSGVRGGRKRPLRPPLVSLARTPLCRRRVGGVDAVLEENDVELRAESQDSAVASG
Px14      DPSDSVSGVRGGRKRPLRPPLVSLARTPLCRRRVGGVDAVLEENDVELRAESQDSAVASG
Mx14      DPSDSVSGVRGGRKRPLRPPLVSLARTPLCRRRVGGVDAVLEENDVELRAESQDSAVASG
Rev2      DPSDSVSGVRGGRKRPLRPPLVSLARTPLCRRRVGGVDAVLEENDVELRAESQDSAVASG
          ************************************************************

WT        PGRIPQPLSGSSGEESATAVEADSTSHDDVHCTCSNDQIITTSIRGLTCDPRMFLRLTHP 300
ΔUL50     PGRIPQPLSGSSGEESATAVEADSTSHDDVHCTCSNDQIITTSIRGLTCDPRMFLRLTHP
Px5       PGRIPQPLSGSSGEESATAVEADSTSHDDVHCTCSNDQIITTSIRGLTCDPRMFLRLTHP
Mx5       PGRIPQPLSGSSGEESATAVEADSTSHDDVHCTCSNDQIITTSIRGLTCDPRMFLRLTHP
Rev1      PGRIPQPLSGSSGEESATAVEADSTSHDDVHCTCSNDQIITTSIRGLTCDPRMFLRLTHP
Px14      PGRIPQPLSGSSGEESATAVEADSTSHDDVHCTCSNDQIITTSIRGLTCDPRMFLRLTHP
Mx14      PGRIPQPLSGSSGEESATAVEADSTSHDDVHCTCSNDQIITTSIRGLTCDPRMFLRLTHP
Rev2      PGRIPQPLSGSSGEESATAVEADSTSHDDVHCTCSNDQIITTSIRGLTCDPRMFLRLTHP
          ************************************************************

WT        ELCELSISYLLVYVPKEDDFCHKICYAVDMSDESYRLGQGSFGEVWPLDRYRVVKVARKH 360
ΔUL50     ELCELSISYLLVYVPKEDDFCHKICYAVDMSDESYRLGQGSFGEVWPLDRYRVVKVARKH
Px5       ELCELSISYLLVYVPKEDDFCHKICYAVDMSDESYRLGQGSFGEVWPLDRYRVVKVARKH
Mx5       ELCELSISYLLVYVPKEDDFCHKICYAVDMSDESYRLGQGSFGEVWPLDRYRVVKVARKH
Rev1      ELCELSISYLLVYVPKEDDFCHKICYAVDMSDESYRLGQGSFGEVWPLDRYRVVKVARKH
Px14      ELCELSISYLLVYVPKEDDFCHKICYAVDMSDESYRLGQGSFGEVWPLDRYRVVKVARKH
Mx14      ELCELSISYLLVYVPKEDDFCHKICYAVDMSDESYRLGQGSFGEVWPLDRYRVVKVARKH
Rev2      ELCELSISYLLVYVPKEDDFCHKICYAVDMSDESYRLGQGSFGEVWPLDRYRVVKVARKH
          ************************************************************
```

```
WT          SETVLTVWMSGLIRTRAAGEQQQPPSLVGTGVHRGLLTATGCCLLHNVTVHRRFHTDMFH 420
ΔUL50       SETVLTVWMSGLIRTRAAGEQQQPPSLVGTGVHRGLLTATGCCLLHNVTVHRRFHTDMFH
Px5         SETVLTVWMSGLIRTRAAGEQQQPPSLVGTGVHRGLLTATGCCLLHNVTVHRRFHTDMFH
Mx5         SETVLTVWMSGLIRTRAAGEQQQPPSLVGTGVHRGLLTATGCCLLHNVTVHRRFHTDMFH
Rev1        SETVLTVWMSGLIRTRAAGEQQQPPSLVGTGVHRGLLTATGCCLLHNVTVHRRFHTDMFH
Px14        SETVLTVWMSGLIRTRAAGEQQQPPSLVGTGVHRGLLTATGCCLLHNVTVHRRFHTDMFH
Mx14        SETVLTVWMSGLIRTRAAGEQQQPPSLVGTGVHRGLLTATGCCLLHNVTVHRRFHTDMFH
Rev2        SETVLTVWMSGLIRTRAAGEQQQPPSLVGTGVHRGLLTATGCCLLHNVTVHRRFHTDMFH
            ************************************************************

WT          HDQWKLACIDSYRRAFCTLADAIKFLNHQCRVCHFDITPMNVLIDVNPHNPSEIVRAALC 480
ΔUL50       HDQWKLACIDSYRRAFCTLADAIKFLNHQCRVCHFDITPMNVLIDVNPHNPSEIVRAALC
Px5         HDQWKLACIDSYRRAFCTLADAIKFLNHQCRVCHFDITPMNVLIDVNPHNPSEIVRAALC
Mx5         HDQWKLACIDSYRRAFCTLADAIKFLNHQCRVCHFDITPMNVLIDVNPHNPSEIVRAALC
Rev1        HDQWKLACIDSYRRAFCTLADAIKFLNHQCRVCHFDITPMNVLIDVNPHNPSEIVRAALC
Px14        HDQWKLACIDSYRRAFCTLADAIKFLNHQCRVCHFDITPMNVLIDVNPHNPSEIVRAALC
Mx14        HDQWKLACIDSYRRAFCTLADAIKFLNHQCRVCHFDITPMNVLIDVNPHNPSEIVRAALC
Rev2        HDQWKLACIDSYRRAFCTLADAIKFLNHQCRVCHFDITPMNVLIDVNPHNPSEIVRAALC
            ************************************************************

WT          DYSLSEPYPDYNERCVAVFQETGTARRIPNCSHRLRECYHPAFRPMPLQKLLICDPHARF 540
ΔUL50       DYSLSEPYPDYNERCVAVFQETGTARRIPNCSHRLRECYHPAFRPMPLQKLLICDPHARF
Px5         DYSLSEPYPDYNERCVAVFQETGTARRIPNCSHRLRECYHPAFRPMPLQKLLICDPHARF
Mx5         DYSLSEPYPDYNERCVAVFQETGTARRIPNCSHRLRECYHPAFRPMPLQKLLICDPHARF
Rev1        DYSLSEPYPDYNERCVAVFQETGTARRIPNCSHRLRECYHPAFRPMPLQKLLICDPHARF
Px14        DYSLSEPYPDYNERCVAVFQETGTARRIPNCSHRLRECYHPAFRPMPLQKLLICDPHARF
Mx14        DYSLSEPYPDYNERCVAVFQETGTARRIPNCSHRLRECYHPAFRPMPLQKLLICDPHARF
Rev2        DYSLSEPYPDYNERCVAVFQETGTARRIPNCSHRLRECYHPAFRPMPLQKLLICDPHARF
            ************************************************************

WT          PVAGLRRYCMSELSALGNVLGFCLMRLLDRRGLDEVRMGTEALLFKHAGAACRALENGKL 600
ΔUL50       PVAGLRRYCMSELSALGNVLGFCLMRLLDRRGLDEVRMGTEALLFKHAGAACRALENGKL
Px5         PVAGLRRYCMSELSALGNVLGFCLMRLLDRRGLDEVRMGTEALLFKHAGAACRALENGKL
Mx5         PVAGLRRYCMSELSALGNVLGFCLMRLLDRRGLDEVRMGTEALLFKHAGAACRALENGKL
Rev1        PVAGLRRYCMSELSALGNVLGFCLMRLLDRRGLDEVRMGTEALLFKHAGAACRALENGKL
Px14        PVAGLRRYCMSELSALGNVLGFCLMRLLDRRGLDEVRMGTEALLFKHAGAACRALENGKL
Mx14        PVAGLRRYCMSELSALGNVLGFCLMRLLDRRGLDEVRMGTEALLFKHAGAACRALENGKL
Rev2        PVAGLRRYCMSELSALGNVLGFCLMRLLDRRGLDEVRMGTEALLFKHAGAACRALENGKL
            ************************************************************
```

```
WT          THCSDACLLILAAQMSYGACLLGEHGAALVSHTLRFVEAKMSSCRVRAFRRFYHECSQTM 660
ΔUL50       THCSDACLLILAAQMSYGACLLGEHGAALVSHTLRFVEAKMSSCRVRAFRRFYHECSQTM
Px5         THCSDACLLILAAQMSYGACLLGEHGAALVSHTLRFVEAKMSSCRVRAFRRFYHECSQTM
Mx5         THCSDACLLILAAQMSYGACLLGEHGAALVSHTLRFVEAKMSSCRVRAFRRFYHECSQTM
Rev1        THCSDACLLILAAQMSYGACLLGEHGAALVSHTLRFVEAKMSSCRVRAFRRFYHECSQTM
Px14        THCSDACLLILAAQMSYGACLLGEHGAALVSHTLRFVEAKMSSCRVRAFRRFYHECSQTM
Mx14        THCSDACLLILAAQMSYGACLLGEHGAALVSHTLRFVEAKMSSCRVRAFRRFYHECSQTM
Rev2        THCSDACLLILAAQMSYGACLLGEHGAALVSHTLRFVEAKMSSCRVRAFRRFYHECSQTM
            ************************************************************

WT          LHEYVRKNVERLLATSDGLYLYNAFRRTTSIICEEDLDGDCRQLFPE 706
ΔUL50       LHEYVRKNVERLLATSDGLYLYNAFRRTTSIICEEDLDGDCRQLFPE
Px5         LHEYVRKNVERLLATSDGLYLYNAFRRTTSIICEEDLDGDCRQLFPE
Mx5         LHEYVRKNVERLLATSDGLYLYNAFRRTTSIICEEDLDGDCRQLFPE
Rev1        LHEYVRKNVERLLATSDGLYLYNAFRRTTSIICEEDLDGDCRQLFPE
Px14        LHEYVRKNVERLLATSDGLYLYNAFRRTTSIICEEDLDGDCRQLFPE
Mx14        LHEYVRKNVERLLATSDGLYLYNAFRRTTSIICEEDLDGDCRQLFPE
Rev2        LHEYVRKNVERLLATSDGLYLYNAFRRTTSIICEEDLDGDCRQLFPE
            ***********************************************
```

Figure S7: Alignments of pUL50, pUL53 and pUL97 of recombinant viruses. Amino acid sequences of reconstituted viruses for pUL50 **(A)** pUL53 **(B)** and pUL97 **(C)**. Phosphosite mutations are highlighted in dark grey, additional mutations in bright grey. * identical amino acids, - no sequence.
